# Antagonistic SMAD2/3 control of TIMP-1, VEGF-A, and hypoxia signaling in myofibroblasts shapes histotype-specific angiogenesis in lung cancer

**DOI:** 10.1038/s41419-026-08677-2

**Published:** 2026-03-30

**Authors:** Natalia Díaz-Valdivia, Paula Duch, Rafael Ikemori, Amelia L. Parker, Marselina Arshakyan, Alejandro Llorente, Alejandro Bernardo, José Rodríguez-Rojas, Josep Lluis Carrasco, Danielle Park, Erik Sahai, Cristina Fillat, Manel Juan, Ernest Nadal, Noemí Reguart, Derek C. Radisky, Oriol Casanovas, Jordi Alcaraz

**Affiliations:** 1 Unit of Biophysics and Bioengineering, Department of Biomedicine, School of Medicine and Health Sciences, Universitat de Barcelona, Barcelona, Spain; 2 Matrix and Metastasis Lab, Cancer Ecosystems Program, Garvan Institute of Medical Research and The Kinghorn Cancer Centre, Darlinghurst, NSW Australia; 3 School of Clinical Medicine, UNSW Medicine and Health, UNSW Sydney, Sydney, NSW Australia; 4 Institute for Bioengineering of Catalonia (IBEC), The Barcelona Institute for Science and Technology (BIST), Barcelona, Spain; 5 Unit of Biostatistics, Department of Basic Clinical Practice, School of Medicine and Health Sciences, Universitat de Barcelona, Barcelona, Spain; 6 Tumour Cell Biology Laboratory, The Francis Crick Institute, London, UK; 7 Institut d’Investigacions Biomèdiques August Pi i Sunyer (IDIBAPS), Barcelona, Spain; 8 Centro de Investigación Biomédica en Red de Enfermedades Raras (CIBERER), Madrid, Spain; 9 Department of Medicine, School of Medicine and Health Sciences, Universitat de Barcelona, Barcelona, Spain; 10 Servei d’Immunologia, Hospital Clinic Barcelona, Barcelona, Spain; 11 Preclinical and Experimental Research in Thoracic Tumors (PRETT), Oncobell, Bellvitge Biomedical Research Institute (IDIBELL), L’Hospitalet de Llobregat, Spain; 12 Department of Medical Oncology, Catalan Institute of Oncology (ICO), L’Hospitalet de Llobregat, Spain; 13 Thoracic Oncology Unit, Hospital Clinic Barcelona, Barcelona, Spain; 14 Department of Cancer Biology, Mayo Clinic, Jacksonville, FL USA; 15 Tumor Angiogenesis Group. ProCURE, Catalan Institute of Oncology, Oncobell, IDIBELL, L’Hospitalet de Llobregat, Spain; 16 Centro de Investigación Biomédica en Red de Enfermedades Respiratorias (CIBERES), Instituto de Salud Carlos III, Madrid, Spain

**Keywords:** Cancer microenvironment, Tumour angiogenesis, Lung cancer, Cancer microenvironment, Tumour angiogenesis

## Abstract

Non-small cell lung cancer (NSCLC) exhibits disparate responses to anti-angiogenic therapies between its two major histologic subtypes, lung adenocarcinoma (LUAD) and squamous cell carcinoma (LUSC), suggesting a histotype-dependent angiogenesis regulation. Tumor-associated fibroblasts (TAFs) exhibit an activated/myofibroblast-like phenotype in NSCLC, and are emerging as major regulators of tumor progression; yet, their role in controlling angiogenesis in NSCLC remains undefined. Here we analyzed angiogenesis/hypoxia markers in NSCLC, and combined transcriptomics (bulk RNA-seq, scRNA-seq), angiogenesis arrays, genetic perturbations and functional in vitro and in vivo assays to dissect the histotype-dependent production of pro-angiogenic factors in TAFs. We observed greater angiogenesis and reduced necrosis/hypoxia in LUAD compared to LUSC across multiple patient cohorts. The LUAD-TAF secretome was primed for angiogenesis through SMAD3-dependent overproduction of key regulators, particularly TIMP-1 and VEGF-A. We also uncovered a novel function for TIMP-1 in promoting endothelial cell hyperbranching over basal VEGF signaling. Conversely, LUSC-TAFs displayed diminished angiogenic effects despite upregulating HIF-1α and a hypoxia-associated transcriptional signature, owing to their lower SMAD3 and compensatory increase in SMAD2. Our study unveils the critical influence of TAFs in shaping the distinct angiogenic landscapes in LUAD and LUSC through the opposing SMAD2/3 regulation of TIMP-1, VEGF-A and hypoxia signaling. These results also highlight the therapeutic potential of targeting stromal SMAD3/TIMP-1 in LUAD or microenvironmental stressors such as hypoxia and acidosis in LUSC. In addition, these findings provide a biological framework for understanding the histotype-dependent patterns of dissemination, immune evasion, and response to anti-angiogenic therapies in NSCLC.

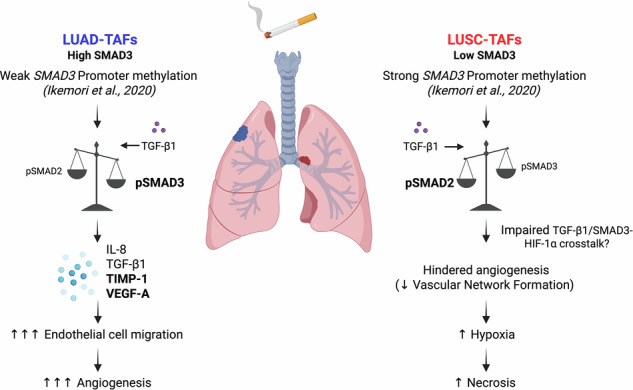

## Introduction

Lung cancer is the leading cause of cancer-related mortality in both men and women [1]. Most patients are diagnosed with non-small cell lung cancer (NSCLC), which is subcategorized into adenocarcinoma (LUAD, ~40% of cases), squamous cell carcinoma (LUSC, ~25%) and other less frequent subtypes. The introduction of immune checkpoint inhibitors (ICI) has revolutionized the therapeutic landscape of lung cancer, yielding durable clinical responses for the first time [2]. However, the long-term beneficial effects of ICI are limited to a minority of patients, indicating the presence of additional immunosuppressive and/or resistance processes [2, 3].

The formation of dysfunctional blood vessels (tumor angiogenesis) [4] has been pointed out as a significant driver of immunosuppression and resistance to immunotherapies and chemotherapies in NSCLC and other highly vascularized desmoplastic tumors [5, 6]. Pathologic angiogenesis, propelled by heightened expression of pro-angiogenic factors like VEGF, may contribute to tumor immune escape by altering the function and/or recruitment of immune cells, and may promote drug resistance by reducing drug penetration into tumors [6, 7]. Consequently, there is growing interest in combining anti-angiogenic agents aiming to normalize tumor vasculature with ICI and/or chemotherapies to overcome resistance and improve patient responses [3, 6, 7]. This strategy has shown promising results in preclinical models in lung cancer and other solid tumors [6, 8], and is being investigated in clinical trials [9–12]. However, clinical experience in NSCLC has been mixed, with drugs such as bevacizumab, ramucirumab and nintedanib demonstrating benefit and receiving approval in LUAD [5, 9, 12, 13] but showing limited efficacy and/or unacceptable toxicity in LUSC [12, 14, 15]. These contrasting outcomes and the limited overall therapeutic benefits attained by anti-angiogenic drugs in LUAD [16] underscore the need for a more nuanced understanding of the histotype-dependent complexity of angiogenesis regulation in NSCLC.

The tumor microenvironment (TME) is increasingly recognized as a key modulator of angiogenesis [4, 17]. Tumor-associated fibroblasts (TAFs) are the most abundant cell type within the TME, and influence angiogenesis through extracellular matrix remodeling and the secretion of angiogenesis-associated factors [4, 18, 19]. In NSCLC, most TAFs exhibit a persistent activated/myofibroblast-like phenotype [20, 21] reminiscent of physiologic programs that promote angiogenesis during tissue repair [17]. Nonetheless, lung TAF activation diverges from that of normal pulmonary fibroblasts, owing in part to the epigenetic reprogramming of the TGF-β pathway [22–24], a major inducer of the myofibroblast phenotype [4]. Recent single-cell techniques and spatial profiling studies have further revealed substantial TAF heterogeneity, including distinct myofibroblast-like states, although none of them have been functionally linked to angiogenesis [21, 25–27]. Despite these advances, it remains unknown how the altered activation of lung TAFs mechanistically impinges on angiogenesis in NSCLC, and whether these mechanisms differ between LUAD and LUSC [27]. Here, we address this gap by dissecting how TAFs regulate angiogenesis in NSCLC. We demonstrate that the epigenetic repression of SMAD3 in LUSC-TAFs elicits a low SMAD3/high SMAD2 state that dampens pro-angiogenic outputs. In contrast, LUAD-derived TAFs exhibit high SMAD3 activity [24] and upregulate key angiogenic mediators, including VEGF-A and TIMP-1. These observations provide a biological framework for the divergent responses of LUAD and LUSC to anti-angiogenic therapies [12, 14, 15] and point to stromal pathways that might be leveraged to enhance vascular normalization and immunotherapy or chemotherapy efficacy.

## Results

### LUAD demonstrates greater angiogenesis and less necrosis/hypoxia than LUSC

**Fig. 1:**
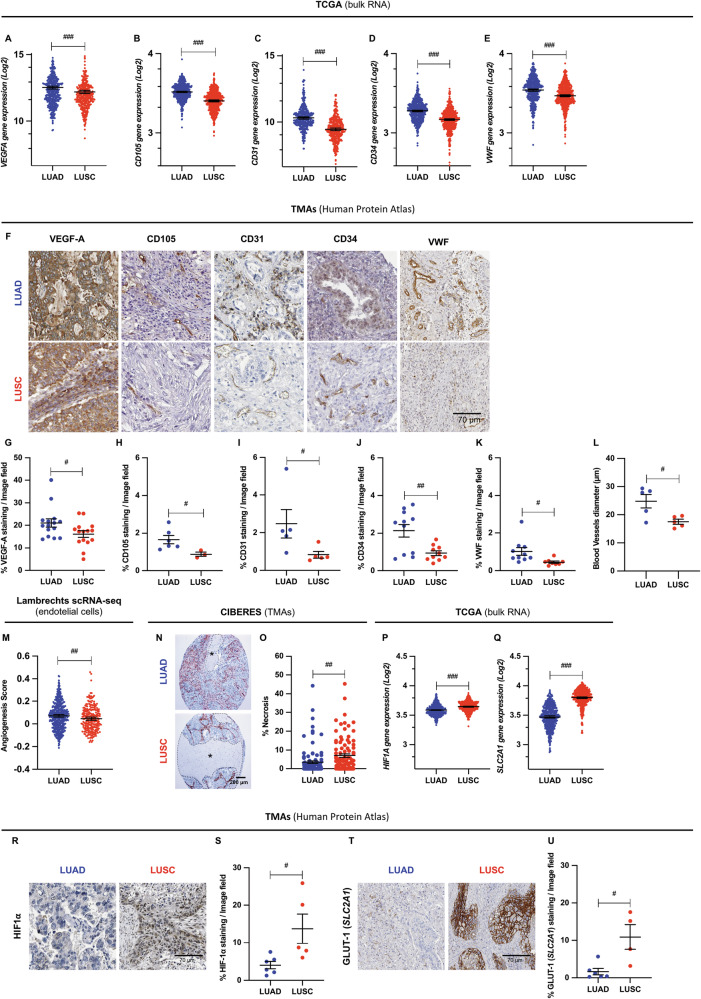
Differential expression of angiogenic and hypoxia/necrosis markers in LUAD and LUSC. Bulk RNA-seq data from the TCGA of angiogenesis genes (*VEGFA* (**A**), *CD105* (**B**)) and endothelial markers (*CD31* (**C**), *CD34* (**D**) and Von Willebrand Factor (*VWF*) (**E**)) in whole-tumor samples (515 LUAD, 501 LUSC). Each dot represents a patient hereafter. **F** Representative zoom images of histologic staining of major angiogenesis (VEGF-A, CD105) and endothelial markers (CD31, CD34, VWF) in TMAs from whole-tumor samples from LUAD and LUSC tumors downloaded from the Human Protein Atlas (HPA) database (6 LUAD; 6 LUSC). Average quantification of the percentage of positively stained area/image for VEGF-A (**G**), CD105 (**H**), CD31 (**I**), CD34 (**J**) and VWF (**K**). **L** Blood vessel diameter quantified from CD31 staining within the HPA TMAs. **M** MSigDb Hallmark Angiogenesis Gene Set scoring of endothelial cells using the Lambrechts scRNAseq dataset [29] (2 LUAD; 2 LUSC). This gene set includes a curated group of 36 genes up-regulated during the formation of blood vessels. **N** Representative histologic images of α-SMA staining of LUAD and LUSC patient samples within TMAs from the CIBERES cohort (112 LUAD, 96 LUSC). Necrotic regions are labeled with *. **O** Percentage of necrotic areas in α-SMA staining within the CIBERES TMAs. RNA-seq data from the TCGA of hypoxia markers *HIF1A* (**P**) and GLUT-1 (*SLC2A1*) (**Q**) in whole-tumor samples. Representative zoom images of histologic staining of major hypoxia markers HIF-1α (**R**) and GLUT-1 (*SLC2A1*) (**T**) from LUAD and LUSC patient samples gathered from the HPA database; quantification of the percentage of positively stained area/image for HIF-1α (**S**) and GLUT-1 (*SLC2A1*) (**U**). Error bars represent mean ± SEM. #, *p* < 0.05; ##, *p* < 0.01; ###, *p* < 0.005, comparing LUAD with LUSC.

To define the angiogenic profiles of LUAD and LUSC, we analyzed bulk tumor RNA-sequencing (RNA-seq) data from the Tissue Cancer Genome Atlas (TCGA), and found higher expression of major angiogenesis (*VEGFA*, *CD105*) and endothelial markers (*CD31*, *CD34*, *VWF*) [28] in LUAD compared to LUSC (Fig. 1A–E). Corresponding histologic staining from the Human Protein Atlas (HPA) confirmed larger positive areas for each marker (Fig. 1F–K) and larger blood vessel lumens (Fig. 1L and Supplementary Fig. 1A; from CD31 images) in LUAD. At the single-cell level, analysis of the MSigDb Hallmark Angiogenesis Gene Set in endothelial cells from the widely-used Lambrechts scRNA-seq dataset [29] showed a significantly higher expression score of this gene signature in LUAD (Fig. 1M). A similar trend was observed with the Zilionis dataset [30] (Supplementary Fig. 1B). Consistent with the link between poor vascularization, hypoxia and necrosis [31], tissue microarrays (TMAs) from the multicentric Spanish CIBERES cohort [20] demonstrated greater necrosis in LUSC (Fig. 1N, O), whereas TCGA (Fig. 1P, Q) and HPA analyses (Fig. 1R–U) revealed elevated expression of the standard hypoxia markers HIF-1α (*HIF1A*) (Fig. 1P, R, S) and GLUT-1 (*SLC2A1*) (Fig. 1Q, T, U) [31] in LUSC. Differences in angiogenesis and necrosis/hypoxia markers between LUAD and LUSC persisted after adjusting TMA and TCGA data for potential confounding clinical variables (stage, age, smoking status and mutation burden when available) (Supplementary Fig. 2A–H). Collectively, these results establish enhanced angiogenesis and reduced hypoxia/necrosis in LUAD compared to LUSC across multiple patient cohorts and methods.

### TAF-endothelial cell interactions are enhanced in LUAD compared to LUSC in tumors and in culture

**Fig. 2:**
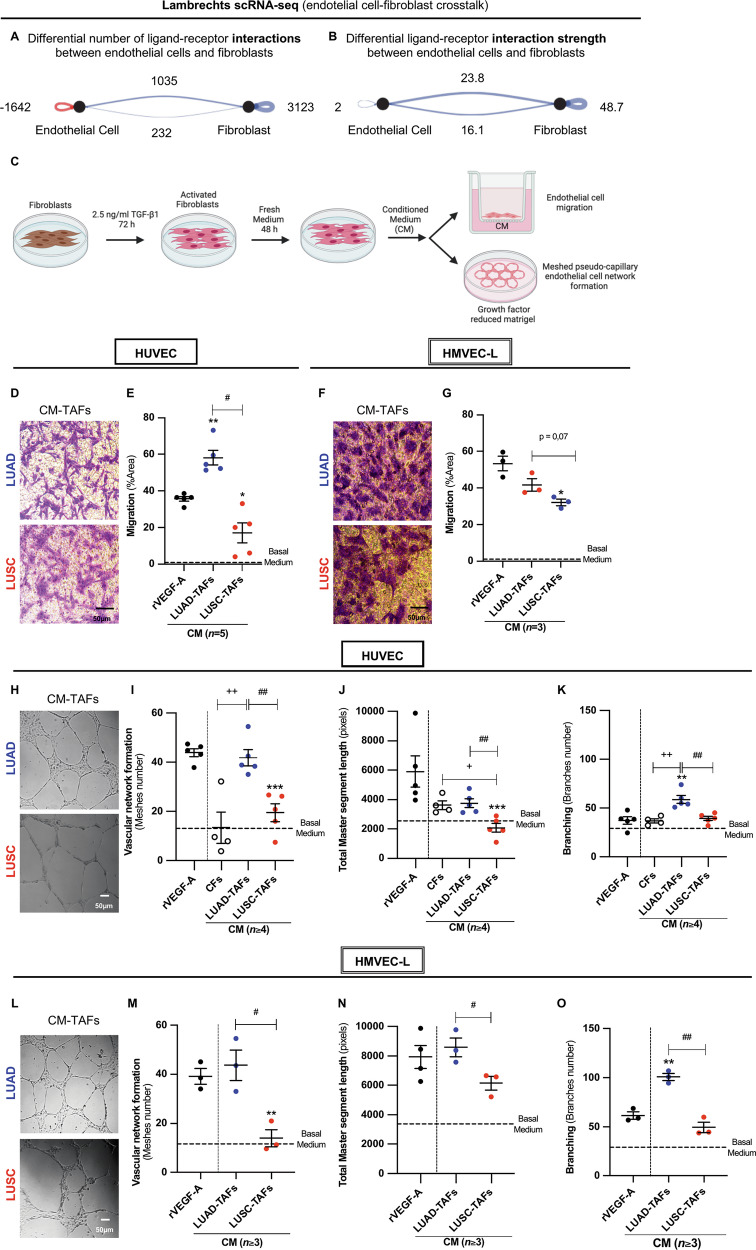
Paracrine endothelial–TAF interactions are upregulated in LUAD versus LUSC in tumors and in culture. CellChat analysis of the heterotypic and homotypic ligand–receptor interactions between endothelial cells and fibroblasts within the Lambrechts scRNA-seq dataset in terms of number of interactions (**A**) and interaction strength (**B**), supporting a role for TAFs as key regulators of endothelial behavior in LUAD through enhanced paracrine signaling. Note that differential scorings correspond to the subtraction LUAD-LUSC (i.e., blue = upregulated in LUAD, red = upregulated in LUSC), and are direction-specific, with top/bottom lines indicating signaling from endothelial cells to fibroblasts (top) and vice versa (bottom lines). **C** Outline of the cell culture experimental design to assess the impact of the conditioned medium (CM) of TGF-β1-activated tumor-associated fibroblasts (TAFs) on either endothelial cell migration (Boyden chamber, top right) or network formation (meshed pseudo-capillary network formation on top of Matrigel, bottom right) (drawings from Biorender hereafter). Representative images of stained HUVEC (**D**) and HMVEC-L (**F**) that have migrated in a Boyden chamber experiment, and corresponding average migration expressed as percentage of stained area upon stimulation with CM of TAFs in HUVEC (5 LUAD, 5 LUSC) (**E**) and HMVEC-L (3 LUAD, 3 LUSC) (**G**). Scale bars indicate 50 µm hereafter. Representative images of HUVEC (**H**) and HMVEC-L (**L**) in a network formation on top of Matrigel assay in response to concentrated CM of TAFs, and corresponding average endothelial cell network descriptors upon stimulation with either human recombinant VEGF-A (rVEGF-A, 50 ng/ml) (positive control) or CM from control fibroblasts (CFs, *n* = 4) and TAFs for HUVEC (5 LUAD, 5 LUSC) (**I**–**K**) and HMVEC-L (≥3 LUAD, ≥3 LUSC) (**M**–**O**), including number of meshes (**I**, **M**), total master segment length (**J**, **N**) and number of branches (**K**, **O**) (described in Supplementary Fig. 12). Error bars represent mean ± SEM. #, *p* < 0.05; ##, *p* < 0.01; ###, *p* < 0.005 comparing LUAD with LUSC. * *p* < 0.05; **, *p* < 0.01; ***, *p* < 0.005, comparing TAFs with rVEGF-A. + *p* < 0.05; ++, *p* < 0.01, comparing TAFs with CFs.

Prompted by the selective pronounced angiogenesis in LUAD, we examined whether TAFs influence this process. For this purpose, we first analyzed the differences in fibroblast–endothelial ligand–receptor interactions in the Lambrechts scRNA-seq dataset [29] by computing differential interaction scores between LUAD and LUSC using the CellChat Package [32]. LUAD showed stronger fibroblast-endothelial cell crosstalk than LUSC, both in number (Fig. 2A) and strength (i.e., probability score) (Fig. 2B) of ligand-receptor interactions, as shown by the higher positive score values and resulting blue color (assigned to LUAD, whereas red labels LUSC samples hereafter). Similar results were obtained with the Zilionis scRNA-seq dataset (Supplementary Fig. 1C, D). This analysis identified over 150 fibroblast-endothelial ligand-receptor pairs significantly upregulated in LUAD versus LUSC in each dataset (Supplementary Table S1A, B). Consistently, an alternative approach (NicheNet package) identified even a larger number of ligand-receptor pairs upregulated in LUAD (Supplementary Table S2 and Supplementary Fig. 3). These single-cell analyses strongly support that TAFs may regulate endothelial behavior in LUAD through enhanced paracrine interactions. To functionally test this hypothesis, we examined the impact of the TAF secretome on endothelial cell migration and meshed pseudo-capillary network formation on Matrigel [33] (Fig. 2C). Since most TAFs exhibit a myofibroblast-like phenotype in NSCLC [20, 21] that is partially lost in culture [34], all TAFs were stimulated with recombinant human TGF-β1 hereafter (unless otherwise indicated), the most prominent myofibroblast-inducing cytokine that is also elevated in NSCLC [35], using a concentration similar to that reported within the bronchoalveolar lavage fluid of patients [36]. Conditioned medium (CM) from both LUAD- and LUSC-TAFs (≥3 LUAD, ≥ 3 LUSC) enhanced endothelial migration in Human Umbilical Vein Endothelial Cells (HUVEC) (Fig. 2D, E) and Human Lung Microvascular Endothelial Cells (HMVEC-L) (Fig. 2F, G) compared to our basal endothelial medium containing a minimal VEGF concentration (2 ng/ml), shown as a horizontal dashed line for reference hereafter, yet this increase was consistently greater in LUAD. Likewise, the CM of LUAD-TAFs elicited an amplified network formation in HUVEC compared to LUSC-TAFs in terms of number of meshes (Fig. 2H, I), total master segment length (Fig. 2J), and number of branches (Fig. 2K), with similar results in HMVEC-L (Fig. 2L–O) (≥3 LUAD, ≥3 LUSC). The number of meshes and total master segment length are indicative of pseudo-capillary network formation, while the branch count is indicative of network tortuosity and pathological disorganization [37]. Remarkably, LUAD-TAFs matched the effects of high-dose (50 ng/ml) recombinant human VEGF-A (rVEGF-A, our positive control [33]) in both HUVEC and HMVEC-L, and surpassed them in branch number (Fig. 2K, O), suggesting the involvement of angiogenic factors other than VEGF-A in branching enhancement. The pro-angiogenic activity of LUAD-TAFs also exceeded that of control fibroblasts (CFs) derived from adjacent uninvolved tissue in the number of meshes and branches in HUVEC (Fig. 2I, K), which was comparable to our basal medium. In contrast, the secretome of LUSC-TAFs induced weaker angiogenic activity than rVEGF-A and comparable or lower activity than CFs in HUVEC (Fig. 2I, J) and HMVEC-L (Fig. 2M, N), yielding the lowest total master segment length (Fig. 2J, N). In control experiments using CM from TAFs not pre-activated with exogenous TGF-β1, LUAD-TAFs robustly promoted both migration and network formation in HUVEC (Supplementary Fig. 4A–D) and HMVEC-L (Supplementary Fig. 4E–H) compared with basal medium, whereas LUSC-TAFs elicited only a modest migratory response and no increase in network formation. Collectively, these results demonstrate that LUAD-TAFs are intrinsically primed to promote angiogenesis, particularly branching, even without exogenous TGF-β1 stimulation, whereas the pro-angiogenic activity of LUSC-TAFs is comparatively reduced.

### Angiogenesis regulators IL-8, TGF-β1, TIMP-1 and VEGF-A are elevated in the LUAD-TAFs secretome compared to LUSC-TAFs

**Fig. 3:**
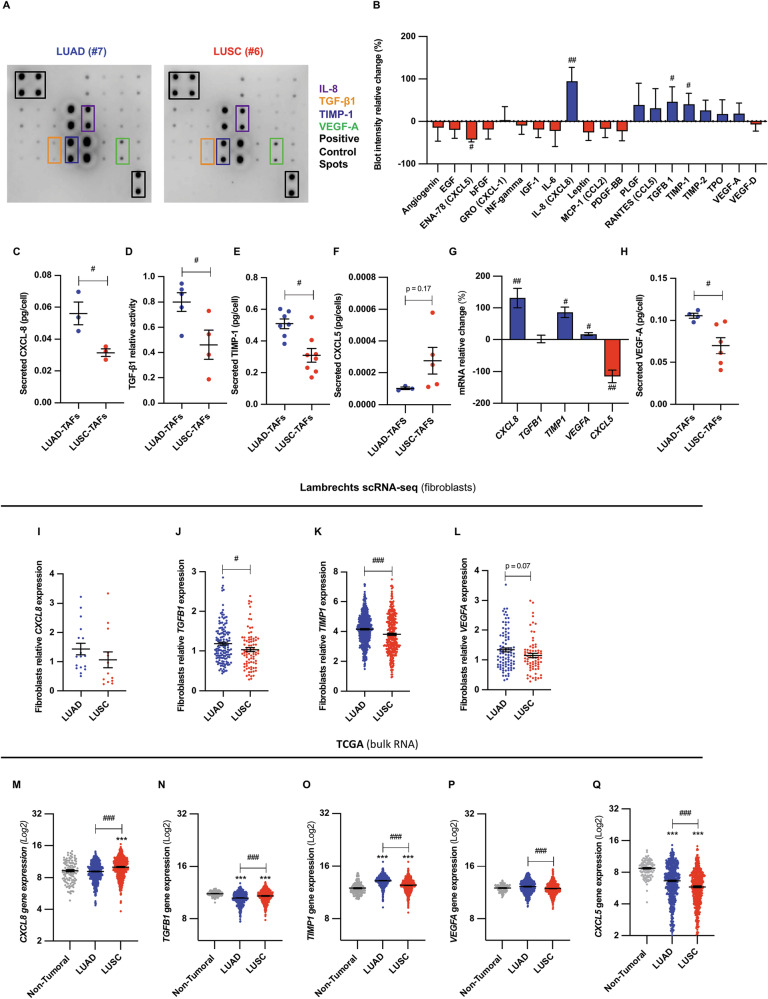
The secretome of LUAD-TAFs exhibits larger expression of pro-angiogenic factors compared to LUSC-TAFs. **A** Representative human angiogenesis antibody dot array analyzing 20 pro-angiogenic factors within the CM of TAFs (additional arrays shown in Supplementary Fig. 5A, B). **B** Average normalized densitometry values for each dot of the CM of LUAD-TAFs with respect to corresponding values of LUSC-TAFs (4 LUAD, 4 LUSC), expressed as relative change (%). Validation of the histotype-dependent secretion of angiogenic regulators IL-8 (**C**), TGF-β1 (**D**), TIMP-1 (**E**) and CXCL5 (**F**) assessed by ELISA or the p(CAGA)_12_ luciferase reporter (**D**) in TAFs (≥3 LUAD, ≥3 LUSC). **G** mRNA expression of the differentially secreted angiogenic factors in LUAD-TAFs and LUSC-TAFs, expressed as relative change (%). **H** ELISA validation of the higher secretion of VEGF-A in LUAD-TAFs compared to LUSC-TAFs (4 LUAD, 6 LUSC). Histotype-expression patterns in fibroblasts within the scRNA-seq Lambrechts dataset of *CXCL8* (**I**), *TGFB1* (**J**), *TIMP1* (**K**) and *VEGFA* (**L**). *CXCL5* expression could not be assessed due to insufficient values (Supplementary Fig. 6A). RNA-seq data from TCGA of *CXCL8* (**M**), *TGFB1* (**N**), *TIMP1* (**O**), *VEGFA* (**P**) and *CXCL5* (**Q**). Error bars represent mean ± SEM. #, *p* < 0.05; ##, *p* < 0.01; ###, *p* < 0.005 comparing LUAD with LUSC. ***, *p* < 0.005, comparing tumor with non-tumoral samples.

Secretome profiling of TGF-β1-activated TAFs (4 LUAD, 4 LUSC) using a 20-factor angiogenesis antibody array (Fig. 3A and Supplementary Fig. 5A, B) revealed significantly higher IL-8, TGF-β1 and TIMP-1 production in LUAD-TAFs, while CXCL5 was selectively upregulated in LUSC-TAFs (Fig. 3B). ELISA and the CAGA-luciferase assay (indicative of bioactive TGF-β1 [24]) confirmed increased IL-8, TIMP-1 and bioactive TGF-β1 in LUAD-TAFs (≥3 LUAD, ≥3 LUSC) (Fig. 3C–F). Transcriptionally, LUAD-TAFs showed elevated *CXCL8* (IL-8) and *TIMP1* mRNA levels, while *TGFB1* levels remained unchanged (Fig. 3G). Given that VEGF-A is transcriptionally regulated by the TGF-β1 transcription factor SMAD3 in normal fibroblasts [38], which we previously showed to be epigenetically repressed in LUSC-TAFs [24], we also examined VEGF-A. LUAD-TAFs exhibited higher VEGF-A mRNA (Fig. 3G) and secreted protein (Fig. 3H), consistent with the array results, although the histotype differences in VEGF-A within the arrays did not reach significance likely due to sample size limitations. Consistently, scRNA-seq analysis of the Lambrechts dataset confirmed upregulation of *CXCL8*, *TGFB1*, *TIMP1* and *VEGFA* (Fig. 3I–L) in LUAD fibroblasts compared to LUSC, whereas *CXCL5* patterns could not be validated due to insufficient values (Supplementary Fig. 6A). Consistent trends were obtained with the Zilionis dataset in most factors (Supplementary Fig. 6F–J). These results support a global transcriptional mechanism underlying the elevated expression of pro-angiogenic factors in LUAD-TAFs. To assess the broader significance of these findings, we analyzed bulk mRNA values of pro-angiogenic factors within the TCGA and found a consistent pattern of higher expression of *TIMP1* and *VEGFA* in LUAD compared to LUSC, whereas other factors exhibited opposite histotype patterns compared to TAFs (Fig. 3M–Q), suggesting that their expression is modulated by other cell types. In line with these findings, our ELISA measurements showed that TIMP-1 and VEGF-A were secreted at markedly higher levels than other factors, suggesting dominant pro-angiogenic roles. In addition, survival analysis revealed that high TIMP-1 and VEGF-A were consistently correlated with poor prognosis in LUAD but not LUSC (Supplementary Fig. 7A–D). Together, these results support that the overproduction of TIMP-1 and VEGF-A in TAFs is a major driver of enhanced angiogenesis in LUAD.

### High SMAD3/low SMAD2 state elicits a pro-angiogenic secretome in control fibroblasts, whereas SMAD3 loss impairs angiogenesis in LUAD-TAFs

**Fig. 4:**
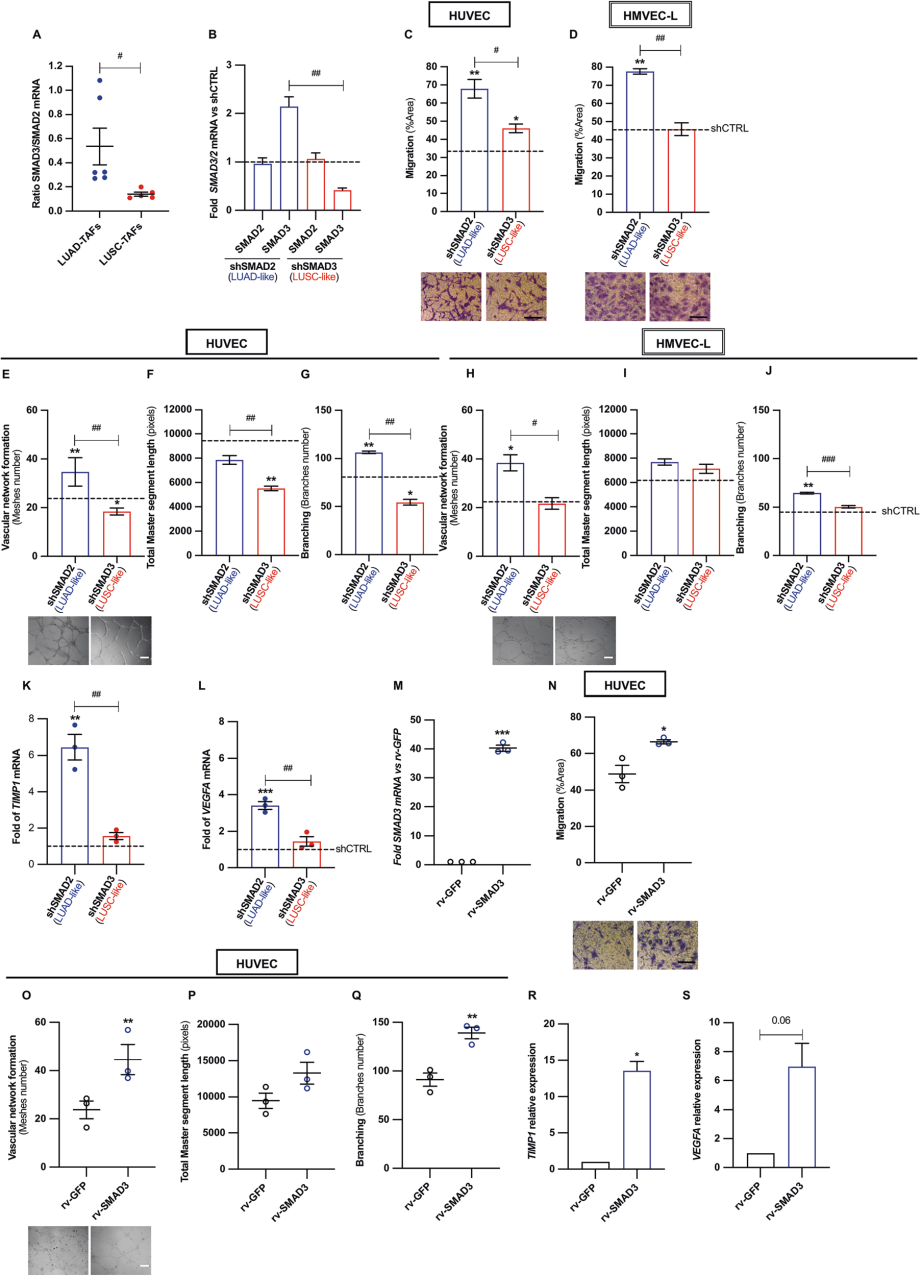
High SMAD3 in control fibroblasts elicits a pro-angiogenic secretome in culture, whereas low SMAD3/high SMAD2 acts otherwise. **A** SMAD3/SMAD2 mRNA ratio in LUAD-TAFs and LUSC-TAFs (6 ADC, 5 SCC) reanalyzed from [24]. **B** Fold *SMAD2* or *SMAD3* mRNA of shSMAD2 (LUSC-like) and shSMAD3 (LUAD-like) CF^hTERT^ (#5) (where the number identifies the selected patient here and thereafter) with respect to shCTRL fibroblasts (dashed line). Endothelial cell migration in HUVEC (**C**) and HMVEC-L (**D**) upon stimulation with the CM of TGF-β1-activated shSMAD2 or shSMAD3 CF^hTERT^ (#5) as described in Fig. 2C. Corresponding values for shCTRL CF^hTERT^ (#5) control shown as a horizontal dashed line hereafter. Endothelial cell network formation descriptors in HUVEC (**E**–**G**) and HMVEC-L (**H**–**J**) elicited by the concentrated CM of shSMAD2 or shSMAD3 CF^hTERT^ (#5) as described in Fig. 2C, including number of meshes (**E**, **H**), total master segment length (**F**, **I**) and number of branches (**G**, **J**). Fold mRNA expression of the pro-angiogenic factors *TIMP1* (**K**) and *VEGFA* (**L**) in shSMAD2 or shSMAD3 CF^hTERT^ (#5) with respect to shCTRL CF^hTERT^ (#5). Additional factors shown in Supplementary Fig. 8A, B. **M** Fold *SMAD3* mRNA of CF^hTERT^ (#5) upon SMAD3 overexpression by lentiviral transduction (rv-SMAD3) with respect to control rv-GFP. **N** Average HUVEC migration upon stimulation with CM of rv-SMAD3 or rv-GFP fibroblasts as in Fig. 2C. Average network descriptors of HUVEC upon stimulation with concentrated CM of rv-SMAD3 or rv-GFP fibroblasts as in Fig. 2C, including number of meshes (**O**), total master segment length (**P**) and number of branches (**Q**). Fold mRNA expression of the pro-angiogenic factors *TIMP1* (**R**) and *VEGFA* (**S**) in rv-GFP or rv-SMAD3 CF^hTERT^ (#5). Mean values of cell culture experiments correspond to *n* ≥ 3 experiments. #, *p* < 0.05; ##, *p* < 0.01; ###, *p* < 0.005 comparing shSMAD2 with shSMAD3. * *p* < 0.05; **, *p* < 0.01; ***, *p* < 0.005 comparing with either shCTRL or rv-GFP.

**Fig. 5:**
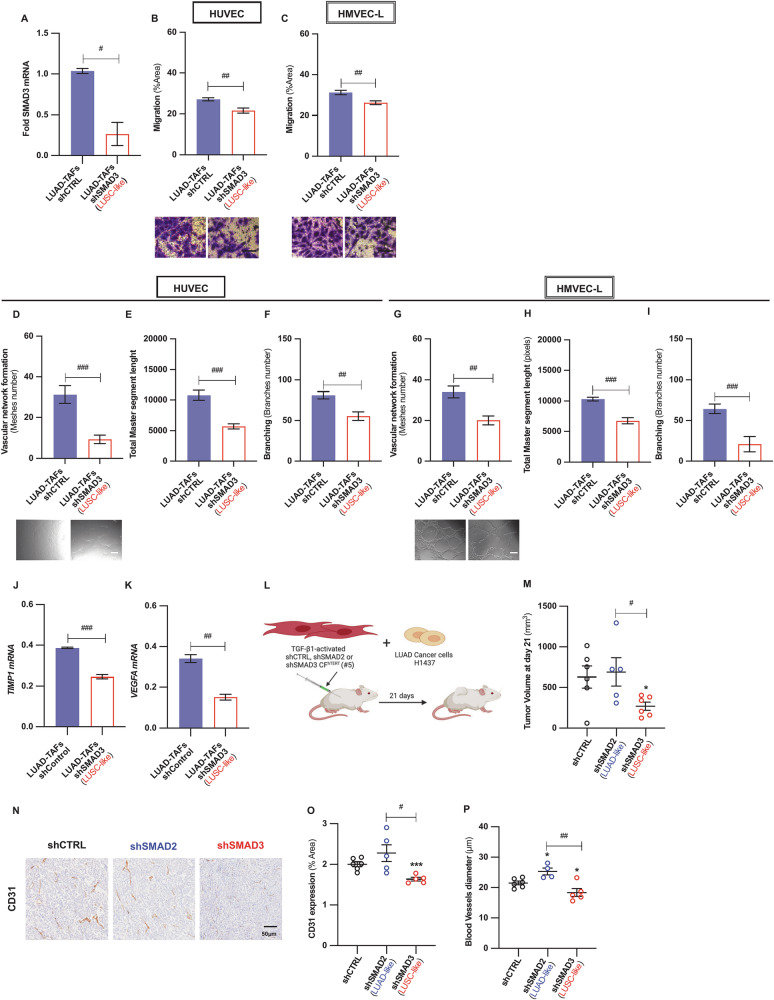
Low SMAD3 impairs angiogenesis in LUAD-TAFs, whereas high SMAD3 or SMAD2 in control fibroblasts elicits a pro- or anti-angiogenic phenotype in vivo. **A** Average fold *SMAD3* mRNA expression in a panel of LUAD-TAFs (*n* = 2: patients #12, #37) upon SMAD3 knockdown by shRNA. Impact of SMAD3 knockdown in LUAD-TAFs (*n* = 2) on the endothelial cell migration (**B**, **C**) and endothelial cell network formation (**D**–**I**) in HUVEC and HMVEC-L, including number of meshes (**D**, **G**), total master segment length (**E**, **H**) and number of branches (**F**, **I**). Corresponding angiogenic downregulation on HUVEC upon knocking down SMAD3 in LUAD-TAFs by siRNA is shown in Supplementary Fig. 9A–D. Impact of SMAD3 knockdown in LUAD-TAFs (*n* = 2) on the mRNA expression of pro-angiogenic factors *TIMP1* (**J**) and *VEGFA* (**K**). Additional factors are shown in Supplementary Fig. 8C, D. **L** Outline of the experimental design used to assess the tumor growth of H1437 LUAD cancer cells subcutaneously co-injected with TGF-β1-activated shCTRL, shSMAD2 or shSMAD3 CF^hTERT^ (#5) (1:2 ratio) into immunodeficient NOD/SCID mice (*n* = 6 mice/condition). **M** Average tumor growth at the end of the observation period for each experimental condition. **N** Representative histologic images of the endothelial marker CD31 in tumors for each experimental condition. Average percentage of CD31-positive area/image field (**O**) and blood vessel diameter (**P**) for each group. Error bars represent mean ± SEM. Mean values of cell culture experiments correspond to *n* ≥ 3 experiments. #, *p* < 0.05; ##, *p* < 0.01; ###, *p* < 0.005 comparing either shSMAD2 with shSMAD3 or LUAD-TAFs shCTRL with shSMAD3. * *p* < 0.05; **, *p* < 0.01; ***, *p* < 0.005 compared with shCTRL.

We previously showed that SMAD3 is upregulated in LUAD-TAFs but suppressed in LUSC-TAFs through DNA promoter hypermethylation, leading to a compensatory increase in SMAD2 and a reduced SMAD3/SMAD2 ratio in LUSC-TAFs [24, 39] (6 LUAD, 5 LUSC; reanalyzed in Fig. 4A). Consistent histotype patterns of the *SMAD3*/*SMAD2* RNA ratio were found in fibroblasts within the Lambrechts and Zilionis scRNA-seq datasets (Supplementary Fig. 6B, K). Accordingly, we examined whether high SMAD3 contributes to the angiogenic priming of LUAD-TAFs. To mimic the histotype patterns of SMAD2/3 in TAFs, we depleted SMAD2 or SMAD3 in hTERT immortalized CFs (#5, where the number identifies the patient hereafter) using shRNA, generating LUAD-like (high SMAD3/low SMAD2) and LUSC-like (low SMAD3/high SMAD2) models, respectively (Fig. 4B) [24, 39]. CM from high SMAD3 fibroblasts (shSMAD2) significantly enhanced endothelial migration (Fig. 4C, D) and network formation (Fig. 4E–J) in HUVEC and HMVEC-L compared to control (shCTRL, dashed horizontal line) or low SMAD3 fibroblasts (shSMAD3), consistent with increased RNA levels of *TIMP1*, *VEGFA* and other angiogenic factors (Fig. 4K, L and Supplementary Fig. 8A, B). Conversely, CM from low SMAD3 fibroblasts elicited the weakest angiogenic responses, resembling LUSC-TAFs. Lentiviral SMAD3 overexpression in CF^hTERT^ (#5) (Fig. 4M) further amplified the pro-angiogenic activity of their secretome (Fig. 4N–Q), and increased *TIMP1* and *VEGFA* (Fig. 4R, S). Conversely, SMAD3 knockdown in LUAD-TAFs (shRNA, *n* = 2) (Fig. 5A) elicited reduced endothelial migration (Fig. 5B, C) and network formation (Fig. 5D–I), along with downregulation of *TIMP1, VEGFA* (Fig. 5J, K) and other pro-angiogenic factors (Supplementary Fig. 8C, D). SMAD3 knockdown by siRNA yielded similar results (Supplementary Fig. 9A–D). Collectively, these findings identify high SMAD3 as a key driver of the pro-angiogenic secretome in LUAD-TAFs and, consequently, its potential as a therapeutic target, while the low SMAD3/high SMAD2 state in LUSC-TAFs confers weaker angiogenic activity.

### Depleting *SMAD3* mRNA in control fibroblasts attenuates tumor growth and angiogenesis in vivo

Our previous work showed that tumor xenografts containing both human fibroblasts and cancer cells recapitulate the selective therapeutic benefits of the anti-angiogenic drug nintedanib in LUAD [40], supporting their utility for studying hystotype-dependent patterns of angiogenesis and responses to anti-angiogenic therapies in NSCLC. Accordingly, we used this approach to test the in vivo pro-angiogenic effects of SMAD2/3 modulation in fibroblasts by co-injecting control (shCTRL), high SMAD3 (shSMAD2), or low SMAD3/high SMAD2 (shSMAD3) CF^hTERT^ (#5) with H1437 LUAD cancer cells into immunocompromised mice (Fig. 5L). After three weeks, tumors bearing low SMAD3 (shSMAD3) fibroblasts showed reduced growth (Fig. 5M), CD31 expression (Fig. 5N, O), and vessel lumen size (Fig. 5P) compared to tumors populated with shCTRL or high SMAD3 (shSMAD2) fibroblasts. In contrast, high SMAD3 fibroblasts promoted the largest and most vascularized tumors (Fig. 5M–P). These results demonstrate that fibroblast SMAD2/3 differentially regulate tumor angiogenesis in vivo.

### In culture and in vivo insights into the influence of TIMP-1 on angiogenesis in LUAD-TAFs

**Fig. 6:**
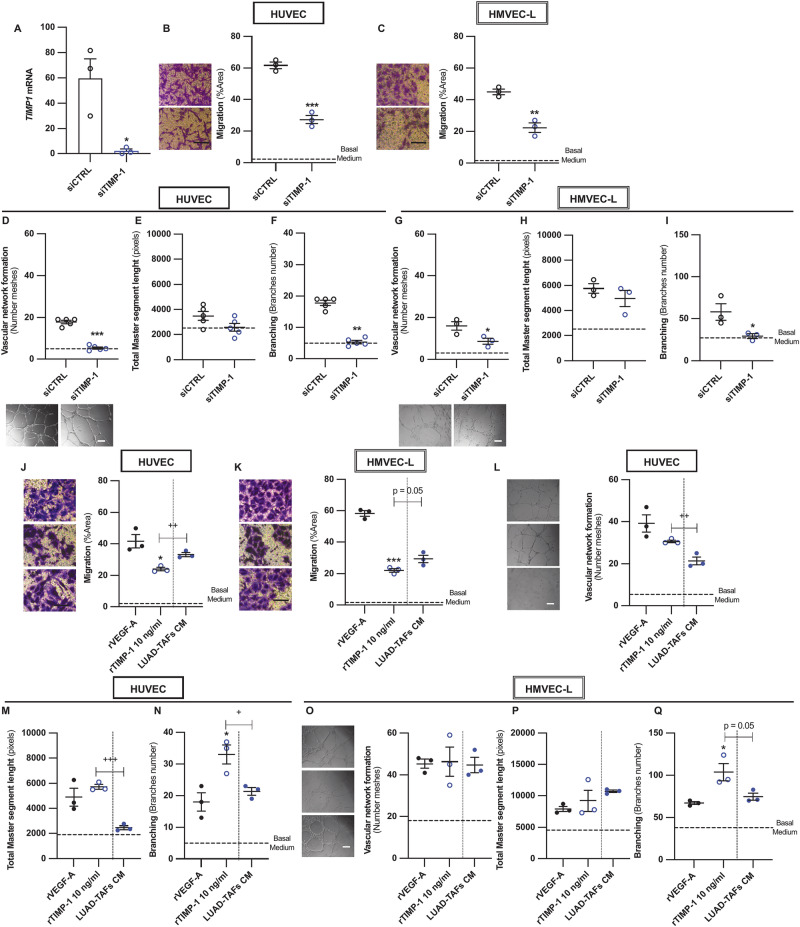
TIMP-1 mediates the hyperbranching pro-angiogenic activity of the secretome of LUAD-TAFs in culture. **A** Average *TIMP1* mRNA expression in a panel of LUAD-TAF^hTERT^ (*n* = 3: patients #7, #12, #37) upon SMAD3 knockdown by siRNA. Impact of TIMP-1 knockdown in LUAD-TAF^hTERT^ (*n* = 3) on the endothelial cell migration (**B**, **C**) and network formation descriptors in HUVEC (**D**-**F**) and HMVEC-L (**G**–**I**) elicited by the concentrated CM of siTIMP-1 compared to siControl (siCTRL) as described in Fig. 2C, including number of meshes (**D**, **G**), total master segment length (**E**, **H**) and number of branches (**F**, **I**). Impact of recombinant VEGF-A (rVEGF-A, 50 ng/ml) (positive control), human recombinant TIMP-1 (rTIMP-1, 10 ng/ml) or the CM of TGF-β1-activated LUAD-TAF^hTERT^ (*n* = 3) on endothelial cell migration (**J**, **K**) and network formation descriptors in HUVEC (**L**–**N**) and HMVEC-L (**O**–**Q**) as described in Fig. 2C. Mean values of cell culture experiments correspond to *n* ≥ 3 experiments. *, *p* < 0.05; **, *p* < 0.01; ***, *p* < 0.005 comparing either siCTRL with siTIMP-1 or rVEGF-A with rTIMP-1. +, *p* < 0.05; ++, *p* < 0.01; +++, *p* < 0.005 comparing rTIMP-1 with the CM of LUAD-TAFs.

Next, we investigated TIMP-1, whose angiogenic effects remain controversial [41, 42], unlike VEGF-A [3]. We first knocked down TIMP-1 in LUAD-TAFs (*n* = 3) using siRNA (Fig. 6A), followed by stimulation with TGF-β1. The secretome of TIMP-1-depleted LUAD-TAFs markedly reduced endothelial cell migration (Fig. 6B, C) compared with controls (siCTRL) in both HUVEC and HMVEC-L, although migration remained above basal medium levels. TIMP-1 depletion in LUAD-TAFs induced an even greater reduction in network formation in both HUVEC and HMVEC-L (Fig. 6D–I), particularly affecting the number of meshes (Fig. 6D, G) and branches (Fig. 6F, I), which were reduced to levels comparable to basal basal medium. Next, we stimulated endothelial cells with recombinant human TIMP-1 (rTIMP-1) at a concentration comparable to its secretion in LUAD-TAFs (10 ng/ml) [43], and found an enhanced migration relative to basal medium, although not to the levels induced by rVEGF-A or LUAD-TAFs (Fig. 6J, K). In contrast, rTIMP-1 consistently enhanced branching in both HUVEC (Fig. 6N) and HMVEC-L (Fig. 6Q), exceeding the effects of rVEGF-A or the CM of LUAD-TAFs. rTIMP-1 also markedly increased other network attributes above basal medium, reaching levels comparable or slightly higher than those induced by our high-dose rVEGF-A control or LUAD-TAFs (Fig. 6L–Q). These results unveil a prominent role of TIMP-1 in promoting angiogenic activity through a hyperbranching phenotype.

**Fig. 7:**
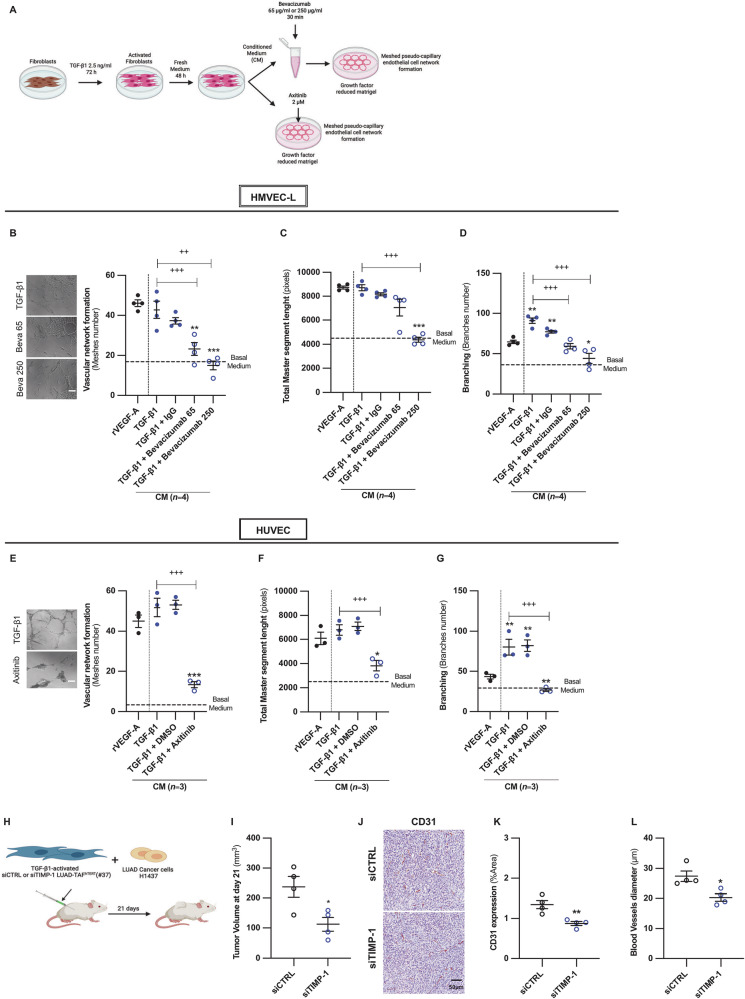
VEGF signaling is required for TIMP-1–mediated pro-angiogenic activity of LUAD-TAFs in culture, while TIMP-1 is necessary for LUAD-TAF–driven tumor angiogenesis in vivo. **A** Outline of the pharmacological cell culture experimental design to assess the requirement of VEGF signaling in the pro-angiogenic priming of the LUAD-TAF secretome rich in TIMP-1. Representative images of HMVEC-L in a network formation assay in response to the CM of LUAD-TAFs (*n* = 4) in the presence of increasing concentrations of bevacizumab (0, 65, 250 µg/ml) (**B**, left panels) or 250 µg/ml IgG control, and corresponding average endothelial cell network descriptors, including number of meshes (**B**, right panel), total master segment length (**C**) and number of branches (**D**). 50 ng/ml rVEGF-A was used as a reference of a strong angiogenic activity, as in Fig. 2 and Fig. 6. Representative images of HUVEC in a network formation assay in response to the CM of LUAD-TAFs (*n* = 3) treated with axitinib (0, 2 µM) (**E**, left panels) or 2 µM DMSO vehicle control, and corresponding average endothelial cell network descriptors, including number of meshes (**E**, right panel), total master segment length (**F**) and number of branches (**G**). 50 ng/ml rVEGF-A was used as an angiogenic reference. **H** Outline of the in vivo study to assess the tumor growth of H1437 LUAD cancer cells subcutaneously co-injected with TGF-β1-activated siCTRL or siTIMP-1 LUAD-TAF^hTERT^ (#37) (1:1 ratio) into immunodeficient NOD/SCID mice (*n* = 4 mice/condition). We previously demonstrated that siRNA-induced downregulation of *TIMP1* mRNA was sustained over a 3-week time-window [43]. **I** Average volume of tumors bearing siCTRL or siTIMP-1 LUAD-TAFs at the end of the observation period. **J** Representative histologic images of the endothelial marker CD31 in tumors for each experimental condition. Average percentage of CD31-positive area/image field (**K**) and blood vessel diameter (**L**) for each group. Error bars represent mean ± SEM. Mean values of cell culture experiments correspond to *n* ≥ 2 experiments. *, *p* < 0.05; **, *p* < 0.01; ***, *p* < 0.005 comparing either each cell culture condition using the CM of LUAD-TAFs with rVEGF-A, or in vivo siCTRL with in vivo siTIMP-1. ++, *p* < 0.01; +++, *p* < 0.005 compared with the corresponding CM of LUAD-TAFs without VEGF inhibitor.

To determine whether rTIMP-1 acts independently of VEGF signaling, we pharmacologically inhibited the VEGF pathway using two clinically-approved compounds: bevacizumab (VEGF-neutralizing antibody) and axitinib (inhibitor of VEGFR-2 and other VEGF receptors), as outlined in Fig. 7A. Inhibition of VEGF signaling consistently abolished the enhanced network formation induced by the secretome of LUAD-TAFs relative to basal medium in either HUVEC or HMVEC-L (Fig. 7B–G) despite the presence of TIMP-1, indicating that TIMP-1 requires a minimal level of VEGF signaling to exert its pro-angiogenic effects. The relevance of TIMP-1 in LUAD angiogenesis was further supported by the identification of *TIMP1*-*CD63* and *TGM2*-*ITGB1* as the only consensus fibroblast-endothelial ligand-receptor pairs significantly upregulated in LUAD, based on the outputs of CellChat and NicheNet applied to two scRNA-seq datasets (Lambrechts, Zilionis) (Supplementary Fig. 10).

To corroborate the pro-angiogenic role of TIMP-1 in vivo, H1437 cancer cells were co-injected with control or TIMP-1-depleted hTERT immortalized LUAD-TAF^hTERT^ (#37) by siRNA for three weeks (Fig. 7H). Tumors harboring TIMP-1-depleted TAFs showed reduced tumor growth (Fig. 7I), CD31 staining (Fig. 7J, K) and blood vessel diameter (Fig. 7L) compared to controls. Collectively, our cell culture and in vivo investigations establish TIMP-1 as a key pro-angiogenic factor in LUAD-TAFs, particularly by promoting a hyperbranching phenotype, in a process that requires basal VEGF signaling and may involve CD63.

### LUSC-TAFs are enriched in a transcriptional signature associated with hypoxia

**Fig. 8:**
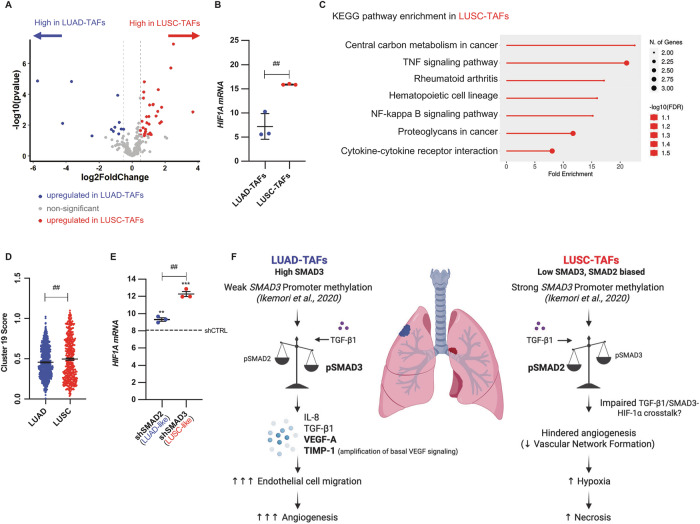
Enrichment of hypoxia transcriptional markers in LUSC-TAFs, and emerging histotype-dependent relationship between SMAD2/3 signaling, angiogenesis and hypoxia in lung TAFs. **A** Volcano plot comparing the differential expression of genes of the hypoxia-associated myofibroblast-like TAF cluster c19 [25] in LUAD-TAFs and LUSC-TAFs assessed by RNA-seq (2 LUAD, 2 LUSC). Further details in Supplementary Table S3. **B** *HIF1A* mRNA levels in lung TAFs (3 LUAD, 3 LUSC). **C** KEGG pathway enrichment analysis of the 36 upregulated genes of the c19 hypoxia-cluster in LUSC-TAFs. **D** Histotype-dependent expression score of the hypoxia-cluster in fibroblasts within the Lambrechts scRNA-seq dataset [29]. **E** Fold mRNA expression of *HIF1A* in shSMAD2 or shSMAD3 CF^hTERT^ (#5) with respect to shCTRL CF^hTERT^ (#5). **F** Summary of the major findings of this study on the histotype-dependent patterns of SMAD2/3 signaling and their relation with angiogenesis/hypoxia regulation in lung TAFs. Error bars represent mean ± SEM. #, *p* < 0.05; ##, *p* < 0.01; ###, *p* < 0.005 comparing LUAD with LUSC or shSMAD2 with shSMAD3. * *p* < 0.05; **, *p* < 0.01; ***, *p* < 0.005 comparing with shCTRL.

Finally, we explored links between the histotype-dependent angiogenesis in NSCLC and the emerging TAF heterogeneity defined by single-cell analyses [25, 26]. To our knowledge, no myofibroblast-like TAF subtypes have been functionally associated with enhanced angiogenesis beyond proximity to blood vessels [44]. In contrast, a recent cross-tissue scRNA-seq study reported a hypoxia-associated myofibroblast cluster c19 [25]. Given the higher necrosis and hypoxia of LUSC versus LUAD tumors (Fig. 1N–U), we asked whether LUSC-TAFs were enriched in the hypoxia-associated cluster c19 (referred to as hypoxia-cluster hereafter). RNA-seq analysis on TAFs showed a higher *SMAD3*/*SMAD2* mRNA ratio and expression of most pro-angiogenic factors in LUAD-TAFs (Supplementary Fig. 11A–G), as expected. Volcano plot analysis further revealed a significant enrichment of 36 hypoxia-cluster genes in LUSC-TAFs (Fig. 8A), including *HIF1A* (HIF-1α gene) (Supplementary Fig. 11H), whereas only 16 were upregulated in LUAD-TAFs (binomial test, *p* < 0.01) (Supplementary Table S3). qRT-PCR confirmed *HIF1A* overexpression in LUSC-TAFs (3 LUAD, 3 LUSC) (Fig. 8B). KEGG pathway enrichment analysis of the 36 upregulated hypoxia-cluster genes in LUSC-TAFs showed a significant abundance of inflammation-associated pathways (Fig. 8C), consistent with smoking-dependent induction of both systemic inflammation [45, 46] and epigenetic repression of SMAD3 in LUSC-TAFs [24]. Similarly, fibroblasts from LUSC tumors showed higher hypoxia-cluster gene expression in the Lambrechts scRNA-seq dataset [29] (Fig. 8D). Aside from hypoxia-related transcriptional changes, analysis of our RNA-seq data in the context of our previously identified consensus ligand-receptor interactions upregulated in LUAD from scRNA-seq data (Supplementary Fig. 11) revealed an opposing upregulation of the *TGM2* ligand in LUSC-TAFs (Supplementary Fig. 11I), arguing against a major role for *TGM2*-*ITGB1*-mediated fibroblast-endothelial crosstalk to the pro-angiogenic priming of LUAD-TAFs.

Mechanistically, the TGF-β pathway is known to enhance HIF-1α expression to amplify hypoxic responses [47], but the contribution of SMAD2/3 to this regulation is poorly understood. Examination of *HIF1A* in shSMAD2 and shSMAD3 CF^hTERT^ (#5) stimulated with TGF-β1 showed higher expression in low SMAD3/high SMAD2 (shSMAD3) conditions (Fig. 8E), supporting that this state promotes hypoxic signaling in LUSC-TAFs. However, despite previous reports in high hypoxic/HIF-1α conditions [48, 49], the increased HIF-1α of LUSC-TAFs (Fig. 8B and Supplementary Fig. 11H) did not upregulate VEGF-A (Fig. 3H, L) or stromal HIF-inducible pro-angiogenic genes (*CXCL12*, *ANGPT2*, *DLL4*) [4], unlike LUAD-TAFs (Supplementary Figs. 6C–E, and 11J–L), supporting an impaired relationship between hypoxia signaling and angiogenesis in LUSC-TAFs. Collectively, our data unveil an unrecognized crucial role for TAFs in driving histotype-dependent patterns of angiogenesis and necrosis/hypoxia in NSCLC, and link these patterns with smoking and subsequent modulation of TGF-β1 signaling through imbalanced SMAD2/3 expression (Fig. 8F).

## Discussion

Previous studies of angiogenesis in NSCLC have been limited by small cohorts or a focus on single histologic subtypes, leaving an incomplete picture of angiogenesis variations between LUAD and LUSC [50–52]. To address these gaps, we used independent datasets and assays (TCGA, HPA, scRNA-seq, and TMAs) to establish that LUAD exhibits more pronounced angiogenesis and larger vessel lumens, whereas LUSC shows enriched hypoxia and necrosis programs. Mechanistically, we revealed an underrecognized major role for TAFs in driving the enhanced angiogenesis in LUAD through a pro-angiogenic secretome dominated by VEGF-A and TIMP-1, as supported by transcriptomics, protein arrays/ELISA, and functional assays. These findings extend prior evidence that TAFs directly instruct tumor angiogenesis [4, 18, 19], supporting a stromal rather than purely cancer cell-derived origin for the vascular phenotype of LUAD. Notably, we identified the TGF-β transcription factor SMAD3 and a high SMAD3/SMAD2 ratio as a central upstream regulator of the pro-angiogenic state of LUAD-TAFs. SMAD3 gain-of-function in control fibroblasts was sufficient to promote angiogenesis, while SMAD3 loss in LUAD-TAFs reduced endothelial migration and network formation in culture, and tumor vascularity in vivo. Consistent with reports in non-tumor fibroblasts and other cell types [38, 53, 54], SMAD3-dependent upregulation of *TIMP1* and *VEGFA* was preserved in LUAD-TAFs, despite epigenetic alterations in the TGF-β/SMAD3 pathway [22]. In line with these observations, previous work in non-tumor cells showed that SMAD3 binds to the VEGFA promoter upon TGF-β1 stimulation [53, 55], and that the TIMP1 promoter contains a SMAD-binding element [56]. Future studies should confirm whether similar promoter interactions occur in LUAD-TAFs.

Of note, our previous work reported the epigenetic repression of SMAD3 in LUSC‑TAFs (driven by tobacco exposure) concomitantly with a compensatory SMAD2 upregulation and resulting reduction in the SMAD3/SMAD2 ratio [24]. Here, we link this altered SMAD2/3 balance to attenuated angiogenic output, despite the hypoxic transcriptome of LUSC-TAFs. These results are consistent with evidence that SMAD2 does not regulate VEGF in fibroblasts [38], although its role in TIMP1 remains uncertain [57, 58]. SMAD2 may even promote anti-angiogenesis through thrombospondin-1 [53], which inversely correlates with microvessel density in LUSC but not LUAD [59], and was upregulated in LUSC-TAFs in our RNA-seq measurements (data not shown). Together, our results unveil the antagonistic functions of SMAD2 and SMAD3 in regulating angiogenesis in lung TAFs, a relationship that has remained poorly defined compared to the established roles of TGF-β-SMAD2/3 in modulating angiogenesis in endothelial and cancer cells [60, 61]. Moreover, they identify the SMAD3/SMAD2 balance as a histotype‑defining stromal switch that tunes angiogenesis in NSCLC, underscoring the need for strategies that overcome current limitations of direct TGF-β1/SMAD3 inhibitors [62] in LUAD.

Single-cell techniques and spatial profiling studies are defining the transcriptional heterogeneity of TAFs, including various myofibroblast-like states [25–27]. Although no TAF subtypes have been functionally linked to enhanced angiogenesis beyond proximity to blood vessels [44], a recent scRNA-seq study identified a hypoxia-associated transcriptional cluster among myofibroblast-like TAFs [25]. This hypoxia-cluster, along with *HIF1A*, was upregulated in LUSC-TAFs compared to LUAD-TAFs. Paradoxically, hypoxia normally induces VEGF-A through HIF-1α [48], yet VEGF-A production remained lower in LUSC-TAFs (and LUSC tumors) compared to LUAD, despite enrichment of *HIF1A* and a hypoxia-associated transcriptional program. This apparent disconnect may reflect the requirement for TGF-β1/SMAD3–HIF‑1α crosstalk to fully engage hypoxia‑induced angiogenic programs [63, 64]: because SMAD3 is epigenetically repressed in LUSC-TAFs [38], HIF-1α‑driven VEGF‑A and other stromal angiogenic factors [63, 64] may be blunted. Consistently, stromal HIF-inducible genes [4] and most LUAD-TAF-specific pro-angiogenic factors were downregulated in LUSC-TAFs. This interpretation reconciles why a hypoxic transcriptional state in LUSC-TAFs does not necessarily translate into high stromal angiogenic activity.

Our study also clarified the debated role of TIMP‑1 in tumor angiogenesis [41, 42]. Genetic loss of TIMP‑1 in LUAD‑TAFs reduced endothelial network formation in culture and tumor vascularization in vivo, whereas recombinant TIMP‑1 promoted endothelial network formation and enhanced branching, a hallmark of tumor vasculature [4, 16]. Notably, inhibiting VEGF signaling completely abrogated the network enhancement elicited by LUAD-TAFs, despite the presence of TIMP-1, revealing that the pro-angiogenic effects of TIMP-1 require basal VEGF signaling. Although the precise mechanisms underlying the pro-angiogenic interaction between VEGF signaling and TIMP-1 remain to be fully elucidated, three lines of evidence implicate CD63. First, our single-cell analyses identified TIMP1-CD63 as a consensus fibroblast-endothelial ligand-receptor pair selectively upregulated in LUAD. Second, previous work reported a prominent pro-angiogenic role for CD63 in endothelial cells, where it forms a functional signaling complex with VEGFR-2 (the dominant endothelial VEGF receptor) that is essential for VEGF-mediated capillary sprouting and tube formation [65]. Third, TIMP-1 in LUAD-TAFs interacts with CD63 in cancer cells to drive tumor progression selectively in LUAD [43]. Collectively, these findings support a model in which VEGF-A-driven angiogenesis is amplified by TIMP-1 by facilitating CD63-VEGFR-2 interactions and downstream signaling. In line with this amplification model, addition of rTIMP-1 to our basal medium containing minimal VEGF enhanced network formation to a level similar to high-dose VEGF-A in the absence of TIMP-1. Consistently, TIMP-1 has been shown to promote VEGF-induced neovascularization in the murine retina [66]. Regardless of the specific mechanisms, our results document a novel endothelial hyperbranching‑specific function of TIMP‑1 in LUAD-TAFs that amplifies VEGF‑A–driven angiogenesis [4, 18, 19], and helps explain why high stromal TIMP‑1 correlates with poor clinical outcomes [67]. These findings also identify stromal TIMP-1 as an attractive therapeutic target in LUAD. While direct clinical inhibitors are not yet available, aberrant TIMP-1 signaling could be potentially modulated indirectly by limiting SMAD3‑dependent TIMP‑1 induction, or by emerging antibody-based strategies [68, 69].

The contrasting angiogenic profiles of LUAD- and LUSC-TAFs provide a biological framework for understanding the histotype-dependent patterns of three clinically-relevant processes in NSCLC: response to anti-angiogenic drugs, tumor dissemination, and immune evasion. The diminished angiogenic capacity of LUSC tumors and LUSC-TAFs may underlie the poor outcomes with anti-angiogenic drugs in LUSC used either as monotherapy or in combination with chemotherapy [9, 14, 15]. Conversely, the elevated expression of pro-angiogenic and immunosuppressive factors in LUAD-TAFs, including VEGF‑A, TGF‑β1 and possibly TIMP‑1 [3, 18, 70], supports better responses to anti-angiogenic strategies and their rational combinations with ICI [9, 11, 13]. Our findings further support testing combinations that concurrently dampen stromal SMAD3 activity and VEGF‑A/TIMP‑1 outputs alongside ICI to improve perfusion and reduce immunosuppression in LUAD. Moreover, because angiogenesis facilitates tumor dissemination [52], the enhanced angiogenesis in LUAD provides a straightforward mechanism for its greater propensity for early metastasis compared with LUSC [71]. Additionally, the higher abundance of tumor endothelial cells in LUAD may contribute to immune evasion by upregulating inhibitory ligands such as PD-L1 and FasL [3, 49], consistent with PD-L1 more accurately predicting long-term ICI benefits in LUAD than in LUSC [72], and with improved responses to ICI combined with anti-VEGF therapies in non-LUSC patients [9, 11, 13]. In contrast, our data support a model in which immunosuppression in LUSC arises predominantly from hypoxia- and necrosis-associated processes, including acidosis and neutrophil recruitment [3, 73, 74]. This recruitment is also enhanced by CXCL5 [75], which was selectively upregulated in LUSC-TAFs, suggesting that therapies targeting the aberrant hypoxic/acidic TME and possibly stromal CXCL5 [31, 75, 76] may be particularly beneficial for LUSC.

There is growing interest in identifying TAF-informed biomarkers for patient stratification to guide cancer therapeutics [27]. Our findings support that stromal SMAD3/SMAD2 ratio, the levels of VEGF‑A and TIMP‑1, and TAF hypoxia-cluster scores from single-cell analyses [24, 25, 40] could guide patient selection for stromal- and vascular-targeted therapeutic combinations in both LUAD and LUSC. However, larger cohorts and prospective biomarker‑driven studies will be needed to establish clinically relevant thresholds. Likewise, the genomic interactions between SMAD3 and VEGFA/TIMP1, the SMAD3–HIF‑1α crosstalk and the SMAD2‑biased anti‑angiogenic programs require further dissection in lung TAFs, as do the mechanisms underlying the VEGF-dependent hyperbranching phenotype elicited by TIMP-1 through CD63 or other processes.

In summary, our study illuminates the histotype‑dependent stromal control of NSCLC angiogenesis, contrasting SMAD3‑activated LUAD‑TAFs with VEGF‑A/TIMP‑1 effector outputs, versus SMAD3‑repressed, SMAD2-biased and hypoxia‑adapted LUSC‑TAFs that provide weaker angiogenic support. This framework reconciles clinical response patterns to anti‑angiogenics and suggests testable, TAF‑guided therapeutic strategies to improve outcomes in this heterogeneous disease: vascular normalization combined with ICI in LUAD (by targeting stromal SMAD3 and/or TIMP‑1) and reversion of microenvironmental conditions like hypoxia and acidosis in LUSC.

## Methods

### Tissue samples and primary fibroblasts

TMAs from NSCLC patients (112 LUAD, 96 LUSC; CIBERES cohort) were analyzed for necrosis (clinicopathologic variables in Supplementary Table S4). Primary fibroblasts were derived as tissue explants from both tumors and paired uninvolved lung tissue (used as control fibroblasts (CFs)) of 22 NSCLC patients (11 LUAD, 11 LUSC; details in Supplementary Material; clinical characteristics in Supplementary Table S5) [77]. Written informed consent was obtained from all patients.

### Recombinant human TIMP-1 production

Recombinant full-length rTIMP-1 was obtained as described [78]. In brief, mature secreted full-length rTIMP-1 was obtained by expressing the pTT/TIMP-1 construct transfected into HEK 293E cells, and purifying it with SP-Sepharose chromatography.

### Cell culture and fibroblast immortalization

HUVEC (ATCC, PCS-100-010) and HMVEC-L (Lonza) cells were maintained in endothelial medium (EGM-2) supplemented with growth factors following the manufacturer’s protocols (Lonza, Endothelial growth medium-2 bullet kit #CC-3162), referred to as basal medium, and used up to 4 passages. Fibroblasts were maintained in DMEM [39] and, for selected patients, immortalized with hTERT [24]. Fibroblast experiments were conducted by seeding them on collagen-coated dishes upon stimulation with 2.5 ng/ml TGF-β1 (Miltenyi Biotec) for 3 days unless otherwise indicated. H1437 LUAD cancer cells (ATCC) were cultured in RPMI-based medium [43].

### Knockdown and overexpression of *SMAD3*, *SMAD2* or *TIMP1* in fibroblasts

*SMAD2* and *SMAD3* were stably knocked down with lentiviral vectors from the Sigma MISSION collection as described [24]. Briefly, HEK293T cells (ATCC CRL-3216) were transfected with suitable plasmids, and their supernatant containing lentivirus was filtered and used to transduce hTERT-immortalized fibroblasts. Transduced cells were selected with puromycin (Sigma). Alternatively, SMAD3 was overexpressed in TAFs using lentiviral vectors rv-SMAD3 or rv-GFP as a control, as described [43]. *SMAD3* and *TIMP1* were also transiently knocked down in hTERT-immortalized LUAD-TAFs with Silencer Select pre-designed siRNAs constructs (107876 SMAD3, 12759 TIMP-1) and a suitable control siRNA (4390843 for SMAD3, Silencer Select negative control No.1 siRNA construct for TIMP-1) (Thermofisher Scientific) using Lipofectamine RNAiMAX [24, 43].

### Conditioned medium

CM from TGF-β1-activated fibroblasts was obtained as described [79]. In brief, 6 × 10^5^ fibroblasts were seeded in a 75 cm^2^ tissue culture flask coated with 0.1 mg/ml collagen type I solution and activated with 2.5 ng/ml TGF-β1 for 72 h in serum-free fibroblast medium. Next, culture medium was washed and replaced with fresh serum-free culture medium for 48 h, and the corresponding CM was collected and stored at –80 °C until use. Its associated cell density was assessed as cells/mL for ELISA normalization [43]. In some experiments, the CM was concentrated 40 times using an Amicon Ultra-2 Centrifugal Filter (Millipore).

### Endothelial cell migration assay

Migration was assessed by Boyden Chamber assay [80]. In brief, 1 × 10^6^ endothelial cells were seeded in endothelial medium for 24 h in serum, serum starved overnight, trypsinized and 1 × 10^5^ cells were subsequently seeded on the upper side of a Transwell insert membrane (6.5-mm diameter, 8-mm pore size; Transwell Costar). 500 μl of CM from TAFs was added to the Transwell bottom compartment to stimulate migration. After 16 h, inserts were removed, washed, and cells that had migrated to the lower side of the insert membrane were stained with 0.1% crystal violet in 2% ethanol and counted in an inverted microscope.

### Endothelial meshed pseudo-capillary network formation assay

Endothelial tube formation was performed as described [33]. Cells seeded on growth factor-reduced Matrigel-coated 24-well plates (Corning; 3 × 10^4^ cells per well) in serum-free medium were stimulated with 2.5 μl of 40× concentrated CM for 16 h, imaged and analyzed using the ImageJ Angiogenesis Analyzer [33] to quantify the number of meshes, branches and total master segment length (Supplementary Fig. 12). 50 ng/ml rVEGF-A (Gibco) served as a positive control. For VEGF signaling inhibition experiments, the CM from LUAD-TAFs was pre-incubated with increasing concentrations of bevacizumab (65 or 250 µg/ml) or IgG control (250 µg/ml) for 30 min before being added to endothelial cells. Alternatively, endothelial cells were treated with the CM of LUAD-TAF containing increasing concentrations of axitinib (0 or 2 µM) or 2 µM DMSO as a vehicle control.

### Human angiogenesis antibody array

CM angiogenesis-related cytokines were analyzed using the Human Angiogenesis Array C1 (RayBiotech) following the manufacturer’s instructions. In brief, CM from TAFs cells were incubated with array membranes overnight at 4 °C, washed and incubated with the biotinylated detection antibody cocktail. The membranes were washed and further incubated with streptavidin-HRP overnight at 4 °C while shaking. The excess buffer was removed, and the protein spots were detected by chemiluminescence with the addition of the detection buffer while exposing for 2 min. Arrays were imaged with the ChemiDoc imaging system (BioRad, Hercules, CA, USA). Densitometric analysis was performed with Image Lab software (BioRad). Pixel density of each duplicated protein spot was averaged and normalized to an internal positive control. Average normalized values for LUAD-TAFs (X_LUAD_) were compared to those of LUSC-TAFs (X_LUSC_) by computing the relative change RC(%) = 100(1- X_LUAD_/X_LUSC_).

### ELISA

CXCL5, IL-8, TIMP-1, and VEGF-A in 10× concentrated CM were measured using DuoSet Human ELISA kits (R&D Systems) or the ab212163 SimpleStep ELISA (Abcam) for CXCL5 as pg/mL following manufacturer’s instructions, and normalized by the number of cells/mL to assess the final concentration as pg/cell as reported [43].

### qRT-PCR

RNA was extracted and reverse-transcribed as described [39, 79]. mRNA levels of *CXCL8* (IL-8), *TGFB1*, *TIMP1*, *VEGFA* and *SMAD2/3* were assessed by qRT-PCR using Taqman probes, with *POL2R* or *ACTB* as endogenous controls, as 2^–ΔCt^ [24, 43].

### RNA sequencing and bioinformatic analysis

RNA-seq analysis was performed as previously described [81] with Trimmed Mean of M-values (TMM) normalization. Total RNA from LUAD-TAFs and LUSC-TAFs was extracted (RNeasy Plus Micro Kit, Qiagen), libraries prepared (KAPA HyperPrep with RiboErase) and sequenced on Illumina HiSeq 2500 (~30 million reads/sample). Reads were quality-checked (TRIM GALORE!, v0.4.2) and aligned to GRCh38 (STAR v1.3.0) [82]. Differentially expressed genes (DEG) were computed with the R-Bioconductor package DESeq2 (www.bioconductor.org, R version 4.4.3) [83], using a *p*-value of less than 0.05, and visualized with a volcano plot. The different number of upregulated genes in LUAD and LUSC was compared with the binomial exact test. KEGG pathway enrichment analysis of upregulated genes in LUSC-TAFs was conducted at 0.1 FDR using the Pathview package [84] and visualized with the ShinyGO 0.82 tool [85].

### TGF-β1 activity reporter assay

TGF-β1 activity within the CM was determined as described [24].

### Histologic analysis

TMAs (CIBERES cohort) were stained for α-SMA [20]. TMA images of VEGF-A, CD31, CD34, CD105 and VWF were obtained from the Human Protein Atlas (HPA) (6 LUAD; 6 LUSC) for analysis [86]. Primary tumor xenografts were processed [24] and stained for CD31 (Ab182981, Abcam) with hematoxylin counterstaining. Blind image processing and analysis were carried out with ImageJ. Images of angiogenesis/endothelial markers were color deconvoluted, binarized, used to calculate the positive area fraction (%), and subsequently averaged for each patient. Blood vessel diameter was computed from CD31 stainings with ImageJ. The percentage of necrotic area per patient was assessed by manually outlining the necrotic areas in α-SMA images with ImageJ and computing the necrotic area (%)/Total area.

### TCGA bulk RNA data analysis

RNA levels of angiogenesis and endothelial genes (*VEGFA*, *PECAM1* (CD31), *CD34*, *ENG* (CD105) and *VWF*) were analyzed from TCGA as reported [24].

### scRNA-seq analysis

Raw gene expression matrices and cellular metadata (including cell type assignments) from the Lambrechts scRNAseq dataset [29] of NSCLC samples (2 LUAD; 2 LUSC) were obtained and processed as described previously [87] using the Seurat package (v4.0.1). Briefly, cells with fewer than 201 UMIs and over 6000 or below 101 expressed genes, or over 10% UMIs derived from the mitochondrial genome, were removed. Gene expression matrices were normalized to total cellular read count and mitochondrial read count as implemented by Seurat’s Normalize and Scale functions. Variably expressed genes were selected as having a normalized expression between 0.125 and 3, and variance exceeding 0.5. Cell type assignments were obtained from the published metadata [29]. Angiogenesis scores were assigned to endothelial cells using the AddModuleScore function (Seurat package, default settings) and the MSigDb HALLMARK-ANGIOGENESIS gene list (MSigDb package) [88]. Differential expression analysis was performed by the Wilcoxon test corrected for multiple comparisons using the Find Markers function. Ligand receptor interaction analysis was performed using the CellChat package [32], based on a ligand-receptor interaction database consisting of the CellChat database merged with the Matricom database to include both cell-associated and extracellular matrix ligands. Validation of inferred ligand-receptor interactions was performed using NicheNet. An alternative scRNA-seq dataset with a balanced representation of LUAD and LUSC samples (5 LUAD; 2 LUSC) was used for further validation [30].

### Survival analysis

Univariate survival analysis was performed in R (survival and survminer R packages) with gene expression thresholds determined via maximally selected rank statistics (maxstat). Kaplan‑Meier plots with log‑rank p‑values visualized survival associations.

### In vivo tumor formation and angiogenesis

Tumor formation of LUAD H1437 cells mixed with fibroblasts (SMAD2/3 or TIMP-1 downregulated) was assessed in 4–6-week-old male NOD/SCID mice (Janvier) as described [24, 43]. H1437 cells were coinjected subcutaneously with pre-activated fibroblast and tumor growth was monitored [43]. After 21 days, xenografts were collected, formalin-fixed, and paraffin-embedded for analysis.

### Statistical analysis

Two-group comparisons were performed with a two-tailed Student *t* test. Tumor volume data were compared with two-way ANOVA (GraphPad Prism v9.0). Multiple linear regression was used to adjust the TMA and TCGA analysis estimates for confounding variables (stage, age, smoking status and mutation burden). Statistical significance was assumed at *p* < 0.05. All data shown as mean ± SEM.

## Supplementary information

Supplementary Materials and Methods

Supplementary Figures

Supplementary Table S1A

Supplementary Table S1B

Supplementary Table S2

Supplementary Table S3

Supplementary Table S4

Supplementary Table S5

## Data Availability

List of fibroblast-endothelial ligand-receptor pairs upregulated in LUAD compared to LUSC from CellChat and NicheNet analyses of scRNA-seq data are available in Supplementary Tables S1A, B and S2, respectively. RNAseq data for the hypoxia-associated cluster c19 in TAFs are available in Supplementary Table S3. Other datasets generated during the current study are available from the corresponding author on reasonable request.
