# Supplementary material for: Antagonistic SMAD2/3 control of TIMP-1, VEGF-A, and hypoxia signaling in myofibroblasts shapes histotype-specific angiogenesis in lung cancer
Source: Cell Death Dis. 2026 Mar 30;17(1):431. doi: 10.1038/s41419-026-08677-2 (PMC13156306; doi:10.1038/s41419-026-08677-2)
Supplement: Supplementary file 1 — Supplementary Materials and Methods [file 41419_2026_8677_MOESM1_ESM.pdf]

Supplementary Materials

Antagonistic SMAD2/3 control of TIMP-1, VEGF-A, and hypoxia signaling in myofibroblasts shapes histotype-specific angiogenesis in lung cancer

By Díaz-Valdivia et al

METHODS

Tissue samples and primary fibroblasts

Tissue microarrays (TMAs) containing samples from surgical NSCLC patients (112 LUAD, 96 LUSC) previously gathered by the CIBERES/Spanish Bronchogenic Carcinoma Cooperative Group (CIBERES cohort) (1) with the approval of the Ethics Committee of the Fundació Parc Taulí were used for immunohistochemistry and necrosis analysis. TMA patients were Caucasian without radiotherapy before surgery. A summary of patient clinicopathologic variables can be found elsewhere (1) and in Suppl. Table S1. Primary fibroblasts were previously derived from tissue explants from a cohort of 22 NSCLC surgical patients (11 LUAD, 11 LUSC) (2). Fibroblasts were obtained from both tumor and paired uninvolved pulmonary tissue (used as control fibroblasts (CFs)) using protocols approved by the Ethics Committees of the *Hospital Clínic de Barcelona*. The mesenchymal purity of fibroblasts was confirmed by the expression of vimentin and absence of cytokeratin expression (3). Selected patients were male, treatment-naïve, Caucasian, 55 years of age or older, and current smokers. Additional clinical characteristics shown in Suppl. Table S2. Written informed consent was obtained from all patients in all cohorts, and all protocols used were in accordance with the Declaration of Helsinki.

Recombinant human TIMP-1 production

Mature secreted full-length rTIMP-1 was obtained by expressing the pTT/TIMP-1 construct transfected into HEK 293E cells (4), and purifying it with SP-Sepharose chromatography as described (5).

Cell culture and fibroblast immortalization

Human Primary Umbilical Vein Endothelial Cells (HUVEC) (ATCC, PCS-100-010) and human Lung Microvascular Endothelial Cells (HMVEC-L) (Lonza) were maintained in endothelial medium (EGM-2), supplemented with growth factors following manufacturer's protocols (Lonza, Endothelial growth medium-2 BulletKit #CC-3162), and used up to 4 passages. The BulletKit contains EBM 2 medium (#CC-3156) and SingleQuots growth factor supplements (#CC-4176) containing 2%FBS, hFGF-B, VEGF, R3-IGF-1, hEGF, hydrocortisone, ascorbic acid, heparin and gentamicin/amphotericin-B GA-1000. This supplemented endothelial medium was referred to as basal medium throughout the manuscript. Fibroblasts were maintained in high-glucose DMEM culture medium with 1 % ITS and antibiotics as described (6). Fibroblasts were used up to passage 6 to avoid replicative senescence (7). Fibroblasts from randomly selected patients were immortalized by transducing hTERT expression (7), which does not affect their response to TGF-β1 (7). Unless otherwise indicated, fibroblast experiments were conducted by seeding them on collagen-coated dishes upon stimulation with 2.5 ng/ml recombinant human TGF-β1 (Miltenyi Biotec) for 3 days to regain their activated phenotype observed in patient samples [18], which is partially lost in culture (3). This concentration is comparable to the average TGF-β1 concentration reported in the bronchoalveolar lavage fluid of lung cancer patients (8). H1437 LUAD cancer cells (ATCC) were cultured in a RPMI-based culture medium as

described (9). Primary cultures and cell lines were confirmed to be Mycoplasma-free, as reported (7).

Knockdown and overexpression of *SMAD3*, *SMAD2* or *TIMP1* in fibroblasts.

SMAD2 and SMAD3 were stably knocked down with lentiviral vectors from the Sigma MISSION collection as described (7). A nonmammalian targeting shRNA vector was used as control (SHC002). Briefly, HEK293T cells (ATCC CRL-3216) were transfected with suitable plasmids, and their supernatant containing lentivirus was filtered and used to transduce hTERT-immortalized fibroblasts. Transduced cells were selected with puromycin (Sigma) as previously reported (7). Alternatively, SMAD3 was overexpressed in TAFs using lentiviral vectors rv-SMAD3 or rv-GFP as control as described (10). Either SMAD3 or TIMP-1 were transiently knocked down in hTERT-immortalized LUAD-TAFs with Silencer Select pre-designed siRNAs constructs (107876 SMAD3, 12759 TIMP-1) and a suitable control siRNA (4390843 for SMAD3, Silencer Select negative control No.1 siRNA construct for TIMP-1) (Thermofisher Scientific) using Lipofectamine RNAiMAX. 48 h after transfection, culture medium was washed and replaced with serum-free fibroblast culture medium as reported (7,10).

Conditioned medium

The conditioned medium (CM) of TGF-β1-activated fibroblasts was obtained as described in (9). Briefly, 6×10^5 fibroblasts were seeded in a 75 cm^2 tissue culture flask coated with 0.1 mg/ml collagen type I solution and activated with 2.5 ng/ml TGF-β1 for 72 h in serum-free fibroblast medium. Next, culture medium was washed and replaced with fresh serum-free culture medium for 48 h, and the corresponding CM was collected, centrifuged to remove suspended cells and stored at -80 ºC until use. Its associated cell density was assessed as cells/mL for ELISA normalization (10). In some experiments, the CM was concentrated 40-times using Amicon Ultra-2 Centrifugal Filter (Millipore).

Endothelial cell migration assay

Cell migration was evaluated using the standard Boyden Chamber assay as described (11). In brief, 1×10^6 endothelial cells were seeded in endothelial medium for 24h in serum, serum starved overnight, trypsinized and 1×10^5 cells were subsequently seeded on the upper side of a Transwell insert membrane (6.5-mm diameter, 8-mm pore size; Transwell Costar). 500 µl of CM from TAFs was added to the Transwell bottom compartment to stimulate migration. After 16 h, inserts were removed, washed and cells that had migrated to the lower side of the insert membrane were stained with 0.1 % crystal violet in 2 % ethanol and counted in an inverted microscope.

Endothelial meshed pseudo-capillary network formation assay

Our protocol is based on the standard "Endothelial Tube Formation Assay" (12,13). In brief, the conditioned medium (CM) was concentrated 40 times using Amicon Ultra-2 Centrifugal Filter (Millipore) according to manufactures' indications. HUVEC or HMVEC-L cells (maximum of 4 passages) were seeded on top of growth factor-reduced Matrigel-coated plates (Corning; 3×10^4 cells per well) in serum-free medium and 2.5 µl of the concentrated CM was added to each well and incubated for 16 h at 37ºC. Cell cultures were images (5 pictures per condition) and analyzed with the Angiogenesis Analyzer plugin in ImageJ (12), which renders a set of vascular network descriptors, including number of meshes, total master segment length and number of branches (Suppl. Fig. 1). Recombinant Human Vascular Endothelial Cell

Growth Factor (rVEGF-A, 50 ng/ml) (# PHC9394, VEGF-165; Gibco) was used as a positive control. For VEGF signaling inhibition experiments, the CM from LUAD-TAFs was pre-incubated with increasing concentrations of bevacizumab (65 or 250 μg/ml) (bevacizumab biosimilar, Aybintio 729170-H, Samsung Bioepis) or IgG control (250 μg/ml) (DDXCH01P-100, Novus Biologicals) for 30 min before being added to endothelial cells. Alternatively, endothelial cells were treated with the CM of LUAD-TAF containing increasing concentrations of axitinib (0 or 2 μM) (Selleckchem) or 2 μM DMSO as a vehicle control.

Human Angiogenesis Antibody Array

The CM was analyzed for angiogenesis-related proteins using the Human Angiogenesis Antibody Array C1 (RayBiotech, Georgia, GA, USA) according to the manufacturer's instructions. In brief, CM from TAFs cells were incubated with the ready-to-use pre-coated array membranes (blocked in blocking buffer provided with the kit) overnight at 4 ºC on a rocking platform shaker. The array membranes were washed three times (5 min each time) with washing buffer I and II (provided with the kit) to remove any unbound proteins. The membranes were then incubated with the biotinylated detection antibody cocktail for 2 h at room temperature. The membranes were washed 3 times for 5 min each with both washing buffers, and further incubated with streptavidin-HRP overnight at 4 ºC while shacking, and washed again. The excess buffer was removed and the protein spots were detected by chemiluminescence by addition of the detection buffer (from the kit) while exposing for 2 min at room temperature. The arrays were visualized and images captured by a ChemiDoc imaging system (BioRad, Hercules, CA, USA). Densitometric analysis was performed with Image Lab software (BioRad). Pixel density of each duplicated protein spot was averaged and normalized to an internal positive control. Average normalized values for LUAD-TAFs (X_{LUAD}) were compared to those of LUSC-TAFs (X_{LUSC}) by computing the relative change $RC(\%)=100(1- X_{LUAD}/X_{LUSC})$.

ELISA

CXCL5, IL-8, TIMP-1 or VEGF-A secretion was analyzed in 10-times concentrated CM using either the Commercial DuoSet Human ELISA kit (R&D Systems) or the ab212163 SimpleStep ELISA (Abcam) for CXCL5 as pg/mL following manufacturer`s instructions, and normalized by the number of cells/mL to assess the final concentration as pg/cell as reported (10).

qRT-PCR

RNA extraction and reverse transcription were performed as described (6,9). mRNA was assessed by qRT-PCR with the StepOnePlus Real-Time PCR System (Applied Biosystems) using Taqman probes for *CXCL8* (IL-8) (#Hs00174103_m1), *CXCL5* (Hs01099660_g1), *TGFB1* (#Hs00998133_m1), *TIMP1* (#Hs00171558_m1), *VEGFA* (#Hs00900055_m1), *HIF1A* (#Hs00153153_m1) and *POL2R* (#Hs00172187_m1; used as housekeeping gene) and Taqman Master Mix (ThermoFisher). *SMAD2/3* mRNA were assessed as described, using *ACTB* as endogenous control (7). Relative gene expression with respect to the housekeeping gene/endogenous control was assessed as $2^{-\Delta Ct}$ as reported (14).

RNA sequencing and bioinformatic analysis

RNAseq analysis was performed using a slightly modified protocol (15) with Trimmed Mean of M-values (TMM) normalization (16). In brief, total RNA from LUAD-TAFs and LUSC-TAFs was extracted using the RNeasy Plus Micro Kit

(Qiagen, 74034). Libraries were prepared with KAPA HyperPrep with RiboErase, amplified with eight PCR cycles, quality-checked via TapeStation, and sequenced on an Illumina HiSeq 2500 instrument on biological replicates per condition. Sequencing generated ~30 million 75- or 100-bp paired-end reads per sample. Reads were quality-trimmed using TRIM GALORE! (v0.4.2, q=20) and aligned to the human GRCh38 (release-86) transcriptome using STAR (v1.3.0) with RSEM default parameters (17,18). Differentially expressed genes (DEG) were computed with the R-Bioconductor package DEseq2 (www.bioconductor.org, R version 4.4.3) (19), using a p-value of less than 0.05, and visualized with a volcano plot. The different number of upregulated genes in LUAD and LUSC was compared with the binomial exact test. KEGG pathway enrichment analysis of upregulated genes in LUSC-TAFs was conducted at 0.1 FDR using Pathview package (20) and visualized with ShinyGO 0.82 tool (21).

TGF-β1 activity reporter assay

The activity of TGF-β1 within the CM was determined using the TGF-β-inducible $p(CAGA)_{12}$ luciferase reporter as described (7).

Histologic analysis

TMAs from the CIBERES cohort were stained for the fibroblast activation marker α-SMA as described (1). TMA images of NSCLC patients (6 LUAD; 6 LUSC) stained for angiogenesis and endothelial markers VEGF-A, CD31, CD34, CD105 and VWF were downloaded from the Human Protein Atlas database (Version 19.3; http://www.proteinatlas.org) for image analysis (22). Primary tumor xenografts obtained from *in vivo* studies were processed as described (7) and stained for CD31 (Ab182981, Abcam). Nuclei were counterstained with hematoxylin. α-SMA and CD31 stainings were imaged at 7-10 randomly selected fields with an upright microscope (BX43) coupled to a digital camera (DP72, Olympus) using a 10× objective for TMAs and a 20× objective for tumor xenografts. Our blind image processing and analysis were carried out with ImageJ (23). Images of angiogenesis/endothelial markers were color deconvoluted, binarized, used to calculate the positive area fraction (%), and subsequently averaged for each patient. Blood vessel diameter was computed from CD31 stainings with ImageJ. The percentage of necrotic area per patient was assessed by manually outlining the necrotic areas in α-SMA images with ImageJ and computing Necrotic area (%)/Total area. Blood vessel diameter assessment from CD31 staining within the HPA TMAs was validated using an automated microvessel segmentation method adapting an algorithm reported elsewhere (24). In brief, vessel segmentation was performed with MATLAB by region-growing in the HSV color space, using hues of stained endothelial cells as seeds and bright yellow-green-cyan pixels as background stop criteria. Image processing was performed using a tiled workflow operating on 1000 × 1000 pixel tiles with 250 pixel overlap to improve segmentation results. Segmented vessel objects underwent lumen detection to quantify lumen perimeter measured from boundaries and equivalent diameter, assuming circular geometry.

Tissue Cancer Genome Atlas (TCGA) bulk RNA data analysis

The bulk mRNA levels of major angiogenesis and endothelial genes (*VEGFA*, *PECAM1* (CD31), *CD34*, *ENG* (CD105) and *VWF*) in whole-tumor samples were analyzed from TCGA (https://cancergenome.nih.gov/) data as reported (7). In brief, level 3 RNA-seq expression data from tumor (517 LUAD, 501 LUSC) and paired normal pulmonary tissue (59 LUAD, LUSC) was downloaded from Firehose Broad GDAC (http://gdac.broadinstitute.org/). LUAD and LUSC RNASeq by Expectation Maximization (RSEM) normalized gene expression values were quantile normalized

and log2-transformed for differential expression analyses using limma package (25) in R.

scRNAseq analysis

We analyzed the few available scRNA-seq datasets containing multiple cell types (including fibroblasts) and a balanced representation of LUAD and LUSC samples (26,27). Raw gene expression matrices and cellular metadata (including cell type assignments) from the Lambrechts scRNAseq dataset (26) of NSCLC samples were obtained and processed as described previously (28) using the Seurat package (v4.0.1) (29). Briefly, cells with fewer than 201 UMIs and over 6000 or below 101 expressed genes, or over 10% UMIs derived from the mitochondrial genome were removed. Gene expression matrices were normalized to total cellular read count and mitochondrial read count as implemented by Seurat's Normalize and Scale functions. Variably expressed genes were selected as having a normalized expression between 0.125 and 3, and variance exceeding 0.5. Cell type assignments were obtained from the published metadata (26). As a validation dataset, human scRNAseq data from Zilionis (27) was downloaded from GSE127465 as human counts normalized, human cell metadata and human genename datasets. A SeuratObject was created from the downloaded data in R (v4.4.2) and subsequently processed using the Seurat package (v5.3.1). Briefly, cells with fewer than 200 UMIs and over 6000 or below 101 expressed genes, or over 15% UMIs derived from the mitochondrial genome were removed. Gene expression matrices were transformed using Seurat's SCTransform algorithm regressed against the mitochondrial read count. Cell types and tumour histological subtypes were assigned from the metadata published at GSE127465. Statistical comparison of fibroblast expression patterns across key genes of interest in LUAD and LUSC tumour types was performed using the FindMarkers algorithm implementing the wilcox test.

Angiogenesis scores were assigned to endothelial cells using the AddModuleScore function (Seurat package, default settings) and the MSigDb HALLMARK-ANGIOGENESIS gene list (MSigDb package) (30). Differential expression analysis was performed by Wilcoxon test corrected for multiple comparisons using the Find Markers function. Ligand receptor interaction analysis was performed using the CellChat package (31), based on a ligand-receptor interaction database consisting of the CellChat database merged with the Matricom database (32) to include both cell-associated and extracellular matrix ligands.

For validation of ligand-receptor interactions identified by CellChat, the NicheNetr package (v2.2.1.1) (33) was utilized. Human Ligand-receptor networks, ligand target matrix and weighted networks were obtained from zenodo (record 7074291) as rds files. Differentially expressed receptors in endothelial cells from LUAD and LUSC tumours were defined as differentially expressed genes in these cells that overlapped with receptors represented in the NicheNet ligand-target database. Ligand activity was predicted for these receptors using the predict_ligand_activities algorithm, on a background of all genes expressed by fiboblasts, the NicheNet ligand target matrix and potential ligands for these receptors identified within the NicheNet database. Ligands with the highest activities were then filtered to include those ligands that were differentially expressed by fibroblasts in LUAD vs LUSC tumours to infer specific ligand-receptors differentially active between LUAD and LUSC fibroblasts and endothelial cells. Consensus ligand-receptor pairs were identified as ligand-receptor pairs differentially active across both CellChat and NicheNet analyses in both the Lambrechts et al. and Zilionis et al. datasets.

Survival analysis

Survival analysis of selected genes was carried out with survival and survminer R packages. Patient samples were split into two groups (high and low expression) according to optimal cutpoint for each gene determined using the maximally selected rank statistics from the maxstat R package. Kaplan-Meier survival plots with log-rank p-values were obtained for LUAD and LUSC patients.

***In vivo* tumor formation and angiogenesis**

The tumorigenicity of LUAD cancer cells mixed with fibroblasts with either SMAD2/3 or TIMP-1 downregulation was examined in 4- to 6-week-old male NOD/SCID mice (Janvier) using protocols approved by the Animal Care and Ethics Committee of the University of Barcelona as reported (7). Mice were housed under specific pathogen-free conditions at constant ambient temperature (22°C–24°C) and humidity (30%–50%) with a 12-hour light-dark cycle. Mice had access to sterilized food and tap water *ad libitum*. All fibroblasts were preactivated with 2.5 ng/mL TGF-β1 for 3 days before coinjection. H1437 cells (0.5×10^6) were mixed with either control shRNA, shSMAD2 or shSMAD3 CF^{hTERT} (#5) (1×10^6) within 100 μL solution of Matrigel mixed with type I collagen (IAC-50, Koken) (1:1) and coinjected subcutaneously in the dorsal flank of NOD/SCID mice. Alternatively, H1437 cells (1×10^6) were mixed with either preactivated siCTRL or siTIMP-1 $LUAD\text{-}TAF^{hTERT}$ (#37) (1×10^6) and coinjected subcutaneously into NOD/SCID mice using the same protocol. We previously confirmed that *TIMP1* mRNA downregulation by siRNA remains stable in LUAD-TAFs within this time frame (10). Tumor growth was assessed as $0.5\times width^2\times length$ using calipers (34). After 21 days, animals were euthanized and tumor xenografts were collected, formalin fixed, and paraffin embedded for further analysis (7). We used a sample size of at least 4 mice for *in vivo* experiments based on our previous study in which 4-6 animals per experimental group were found to be sufficient to detect significant changes in tumor growth (10) and to address the increasing ethical demand to keep animal use to a minimum.

Statistical analysis

Two-group comparisons were performed with two-tailed Student t-test. Tumor volume data were compared with two-way ANOVA (GraphPad prism v9.0). Statistical significance was assumed at $p < 0.05$. All data shown as mean ± SEM. All R analyses were conducted using R version 4.4.3. P-values were corrected for multiple comparisons as indicated.

REFERENCES

1. Alcaraz J, Carrasco JL, Millares L, Luis IC, Fernández-Porras FJ, Martínez-Romero A, et al. Stromal markers of activated tumor associated fibroblasts predict poor survival and are associated with necrosis in non-small cell lung cancer. Lung Cancer. 2019;135:151–60.

2. Puig M, Lugo R, Gabasa M, Gimenez A, Velasquez A, Galgoczy R, et al. Matrix Stiffening and beta(1) Integrin Drive Subtype-Specific Fibroblast Accumulation in Lung Cancer. Mol Cancer Res. 2015 Jan;13(1):161–73.

3. Vizoso M, Puig M, Carmona FJ, Maqueda M, Velásquez A, Gómez A, et al. Aberrant DNA methylation in Non Small Cell Lung Cancer associated fibroblasts. Carcinogenesis. 2015;36:1453–63.

4. Crombez L, Marques B, Lenormand JL, Mouz N, Polack B, Trocme C, et al. High level production of secreted proteins: example of the human tissue inhibitor of metalloproteinases 1. Biochem Biophys Res Commun. 2005 Nov 25;337(3):908–15.

5. Batra J, Robinson J, Soares AS, Fields AP, Radisky DC, Radisky ES. Matrix metalloproteinase-10 (MMP-10) interaction with tissue inhibitors of metalloproteinases TIMP-1 and TIMP-2: binding studies and crystal structure. J Biol Chem. 2012 May 4;287(19):15935–46.

6. Juste-Lanas Y, Díaz-Valdivia N, Llorente A, Ikemori R, Bernardo A, Arshakyan M, et al. 3D collagen migration patterns reveal a SMAD3-dependent and TGF-β1-independent mechanism of recruitment for tumour-associated fibroblasts in lung adenocarcinoma. Br J Cancer. 2023 Apr;128(6):967–81.

7. Ikemori R, Gabasa M, Duch P, Vizoso M, Bragado P, Arshakyan M, et al. Epigenetic SMAD3 Repression in Tumor-Associated Fibroblasts Impairs Fibrosis and Response to the Antifibrotic Drug Nintedanib in Lung Squamous Cell Carcinoma. Cancer Res. 2020 Jan 15;80(2):276–90.

8. Domagała-Kulawik J, Hoser G, Safianowska A, Grubek-Jaworska H, Chazan R. Elevated TGF-beta1 concentration in bronchoalveolar lavage fluid from patients with primary lung cancer. Arch Immunol Ther Exp Warsz. 2006 Apr;54(2):143–7.

9. Gabasa M, Ikemori R, Hilberg F, Reguart N, Alcaraz J. Nintedanib selectively inhibits the activation and tumor-promoting effects of fibroblasts from lung adenocarcinoma patients. Br J Cancer. 2017;117:1128–38.

10. Duch P, Díaz-Valdivia N, Ikemori R, Gabasa M, Radisky ES, Arshakyan M, et al. Aberrant TIMP-1 overexpression in tumor-associated fibroblasts drives tumor progression through CD63 in lung adenocarcinoma. Matrix Biol. 2022 Aug;111:207–25.

11. Valanti EK, Dalakoura-Karagkouni K, Fotakis P, Vafiadaki E, Mantzoros CS, Chroni A, et al. Reconstituted HDL-apoE3 promotes endothelial cell migration through ID1 and its downstream kinases ERK1/2, AKT and p38 MAPK. Metabolism. 2022 Feb;127:154954.

12. Carpentier G, Berndt S, Ferratge S, Rasband W, Cuendet M, Uzan G, et al. Angiogenesis Analyzer for ImageJ - A comparative morphometric analysis of "Endothelial Tube Formation Assay" and "Fibrin Bead Assay." Sci Rep. 2020 July 14;10(1):11568.

13. Torres P, Díaz J, Arce M, Silva P, Mendoza P, Lois P, et al. The salivary peptide histatin-1 promotes endothelial cell adhesion, migration, and angiogenesis. FASEB J. 2017 Nov;31(11):4946–58.

14. Livak KJ, Schmittgen TD. Analysis of relative gene expression data using real-time quantitative PCR and the 2(-Delta Delta C(T)) Method. Methods. 2001 Dec;25(4):402–8.

15. Park D, Wershof E, Boeing S, Labernadie A, Jenkins RP, George S, et al. Extracellular matrix anisotropy is determined by TFAP2C-dependent regulation of cell collisions. Nat Mater. 2019 Oct 28;

16. Robinson MD, Oshlack A. A scaling normalization method for differential expression analysis of RNA-seq data. Genome Biol. 2010;11(3):R25.

17. Li B, Dewey CN. RSEM: accurate transcript quantification from RNA-Seq data with or without a reference genome. BMC Bioinformatics. 2011 Aug 4;12:323.

18. Dobin A, Davis CA, Schlesinger F, Drenkow J, Zaleski C, Jha S, et al. STAR: ultrafast universal RNA-seq aligner. Bioinforma Oxf Engl. 2013 Jan 1;29(1):15–21.

19. Love MI, Huber W, Anders S. Moderated estimation of fold change and dispersion for RNA-seq data with DESeq2. Genome Biol. 2014;15(12):550.

20. Luo W, Brouwer C. Pathview: an R/Bioconductor package for pathway-based data integration and visualization. Bioinforma Oxf Engl. 2013 July 15;29(14):1830–1.

21. Ge SX, Jung D, Yao R. ShinyGO: a graphical gene-set enrichment tool for animals and plants. Bioinforma Oxf Engl. 2020 Apr 15;36(8):2628–9.

22. Uhlen M, Oksvold P, Fagerberg L, Lundberg E, Jonasson K, Forsberg M, et al. Towards a knowledge-based Human Protein Atlas. Nat Biotechnol. 2010 Dec;28(12):1248–50.

23. Abramoff MD, Magelhaes PJ, Ram SJ. Image processing with ImageJ. Biophotonics Int. 2004;11(7):36:42.

24. Reyes-Aldasoro CC, Williams LJ, Akerman S, Kanthou C, Tozer GM. An automatic algorithm for the segmentation and morphological analysis of microvessels in immunostained histological tumour sections. J Microsc. 2011 June;242(3):262–78.

25. Ritchie ME, Phipson B, Wu D, Hu Y, Law CW, Shi W, et al. Limma powers differential expression analyses for RNA-sequencing and microarray studies. Nucleic Acids Res. 2015;43(Epub):e47.

26. Lambrechts D, Wauters E, Boeckx B, Aibar S, Nittner D, Burton O, et al. Phenotype molding of stromal cells in the lung tumor microenvironment. Nat Med. 2018 Aug;24(8):1277–89.

27. Zilionis R, Engblom C, Pfirschke C, Savova V, Zemmour D, Saatcioglu HD, et al. Single-Cell Transcriptomics of Human and Mouse Lung Cancers Reveals Conserved Myeloid Populations across Individuals and Species. Immunity. 2019 May 21;50(5):1317-1334.e10.

28. Parker AL, Bowman E, Zingone A, Ryan BM, Cooper WA, Kohonen-Corish M, et al. Extracellular matrix profiles determine risk and prognosis of the squamous cell carcinoma subtype of non-small cell lung carcinoma. Genome Med. 2022 Nov 21;14(1):126.

29. Hao Y, Hao S, Andersen-Nissen E, Mauck WM, Zheng S, Butler A, et al. Integrated analysis of multimodal single-cell data. Cell. 2021 June 24;184(13):3573-3587.e29.

30. Subramanian A, Tamayo P, Mootha VK, Mukherjee S, Ebert BL, Gillette MA, et al. Gene set enrichment analysis: a knowledge-based approach for interpreting genome-wide expression profiles. Proc Natl Acad Sci U S A. 2005 Oct 25;102(43):15545–50.

31. Jin S, Plikus MV, Nie Q. CellChat for systematic analysis of cell-cell communication from single-cell transcriptomics. Nat Protoc. 2025 Jan;20(1):180–219.

32. Lamba R, Paguntalan AM, Petrov PB, Naba A, Izzi V. MatriCom: a scRNA-Seq data mining tool to infer ECM-ECM and cell-ECM communication systems. BioRxiv Prepr Serv Biol. 2024 Dec 16;2024.12.10.627834.

33. Browaeys R, Saelens W, Saeys Y. NicheNet: modeling intercellular communication by linking ligands to target genes. Nat Methods. 2020 Feb;17(2):159–62.

34. Gabasa M, Radisky ES, Ikemori R, Bertolini G, Arshakyan M, Hockla A, et al. MMP1 drives tumor progression in large cell carcinoma of the lung through fibroblast senescence. Cancer Lett. 2020 in 2nd revision;
