# Supplementary material for: Antagonistic SMAD2/3 control of TIMP-1, VEGF-A, and hypoxia signaling in myofibroblasts shapes histotype-specific angiogenesis in lung cancer
Source: Cell Death Dis. 2026 Mar 30;17(1):431. doi: 10.1038/s41419-026-08677-2 (PMC13156306; doi:10.1038/s41419-026-08677-2)
Supplement: Supplementary file 2 — Supplementary Figures [file 41419_2026_8677_MOESM2_ESM.pdf]

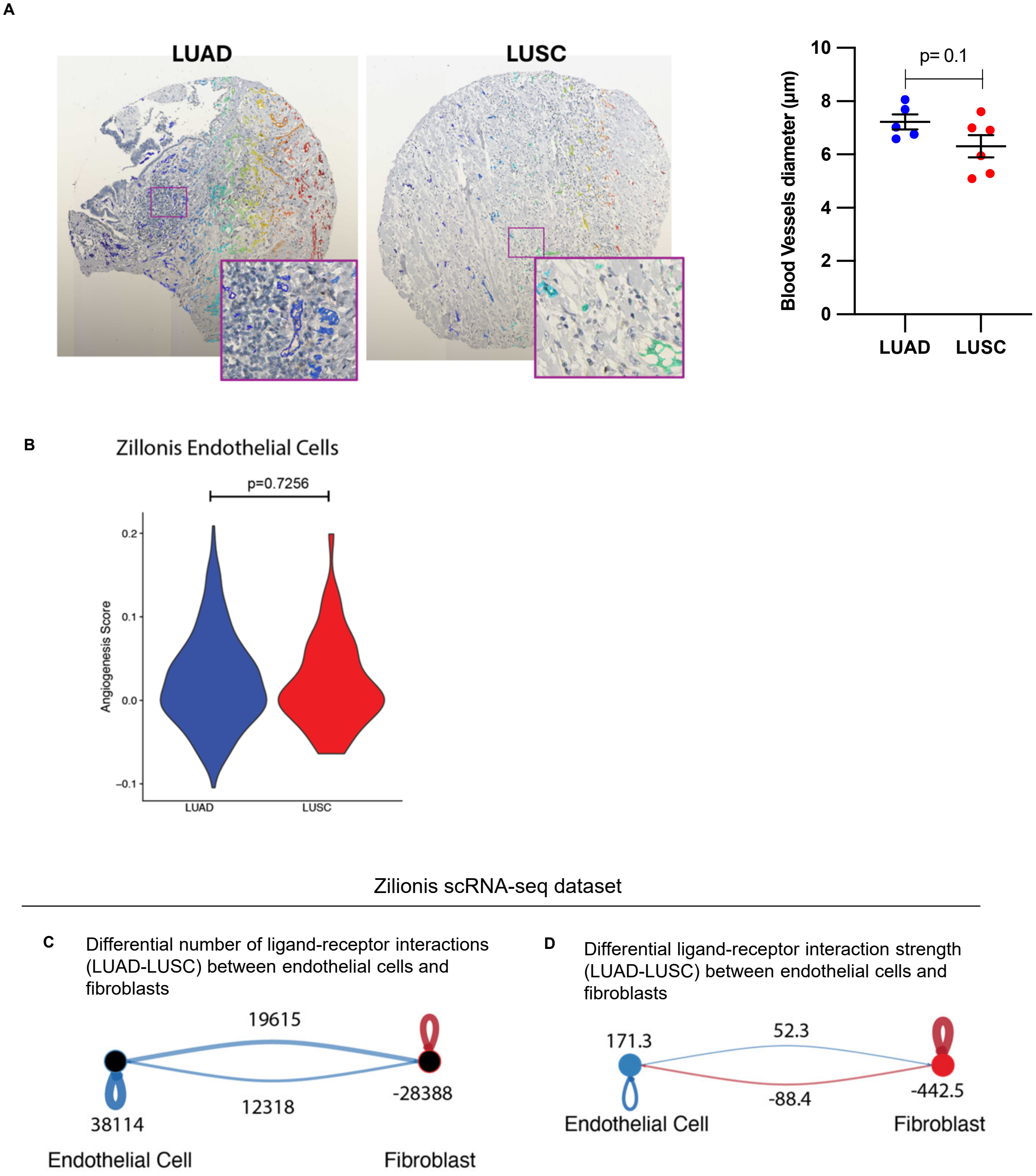

Supplementary Figure 1. Histotype-dependent patterns of angiogenesis (A-B) and endothelial cell-fibroblast interactions (C-D). (A) Blood vessel diameter quantified from CD31 staining within the HPA TMAs using an automated microvessel segmentation method adapting an algorithm reported elsewhere (Reyes-Aldasoro et al, J Microsc 2011, PMID: 21118252). In brief, vessel segmentation was performed with MATLAB by region-growing in the HSV color space, using hues of stained endothelial cells as seeds and bright yellow-green-cyan pixels as background stop criteria. Image processing was performed using a tiled workflow operating on 1000 × 1000 pixel tiles with 250 pixel overlap to improve segmentation results. Segmented vessel objects underwent lumen detection to quantify lumen perimeter measured from boundaries and equivalent diameter, assuming circular geometry. Representative segmented vessels are shown on the left for LUAD and LUSC, and corresponding quantification is shown on the right. (B) MSigDb Hallmark Angiogenesis Gene Set scoring of endothelial cells using the Zilionis scRNAseq dataset (5 LUAD, 2 LUSC) (Zilionis et al, Immunity 2019; PMID: 30979687). (C-D) CellChat analysis of the heterotypic and homotypic ligand–receptor interactions between endothelial cells and fibroblasts within the Zilionis scRNA-seq dataset in terms of number of interactions (C) and interaction strength (D) as in Fig 2A-B.

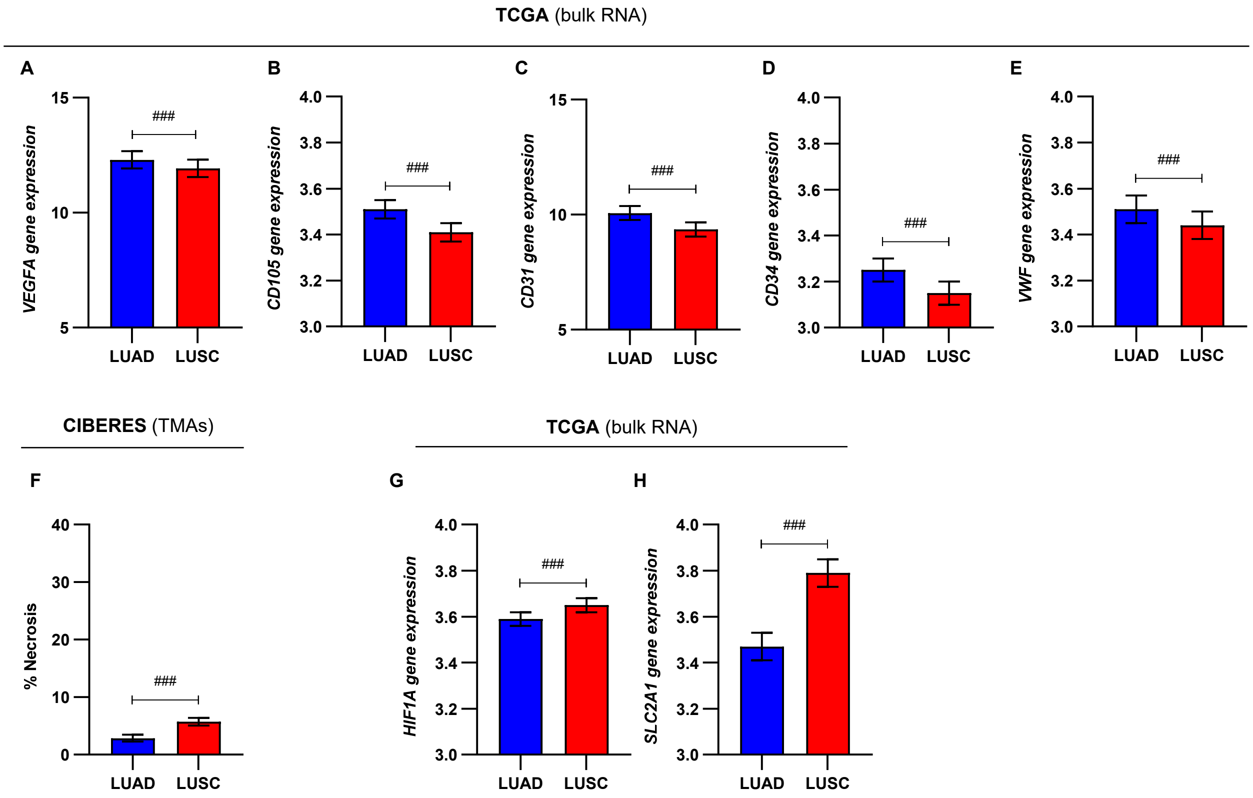

Supplementary Figure 2. **Differential expression of angiogenic and hypoxia/necrosis markers in LUAD and LUSC adjusted for potential confounders (stage, age, smoking status and mutation burden when available) using multiple linear regression.** (A-E) Bulk RNA-seq data from the TCGA of angiogenesis genes (VEGFA (A), CD105 (B)) and endothelial markers (CD31 (C), CD34 (D) and Von Willebrand Factor (VWF) (E)) in whole-tumor samples. (F) Percentage of necrotic areas in α-SMA staining within the CIBERES TMAs. (G-H) RNA-seq data from the TCGA of hypoxia markers HIF1A (G) and GLUT-1 (SLC2A1) (H) in whole-tumor samples. Error bars represent mean ± SEM. #, $p < 0.05$; ##, $p < 0.01$; ###, $p < 0.005$ comparing LUAD with LUSC.

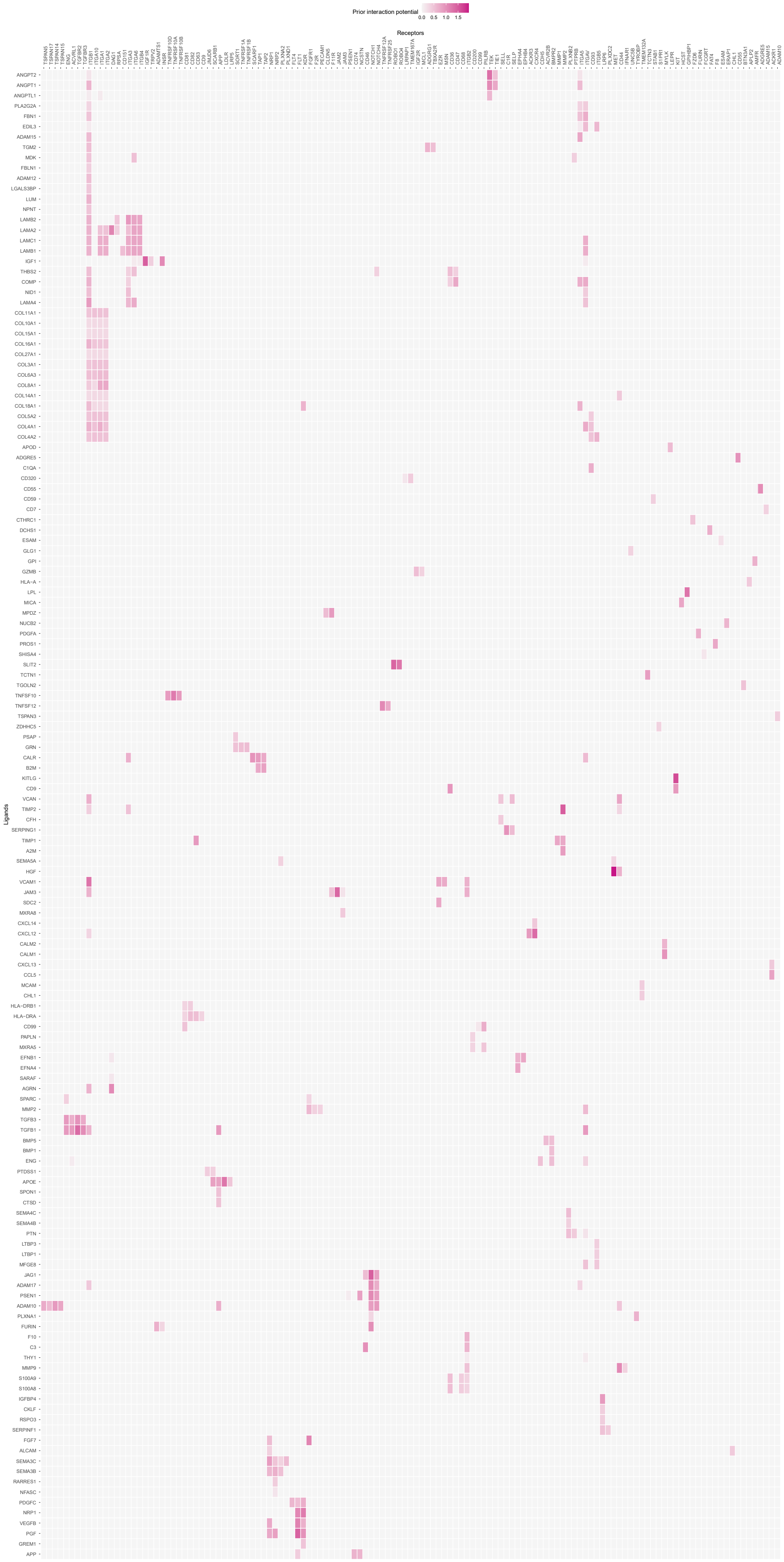

Supplementary Figure 3. Heatmap of fibroblast-endothelial cell (EC) ligand-receptor pairs upregulated in LUAD compared with LUSC identitifed using NicheNet in the Lambrechts scRNA-seq dataset.

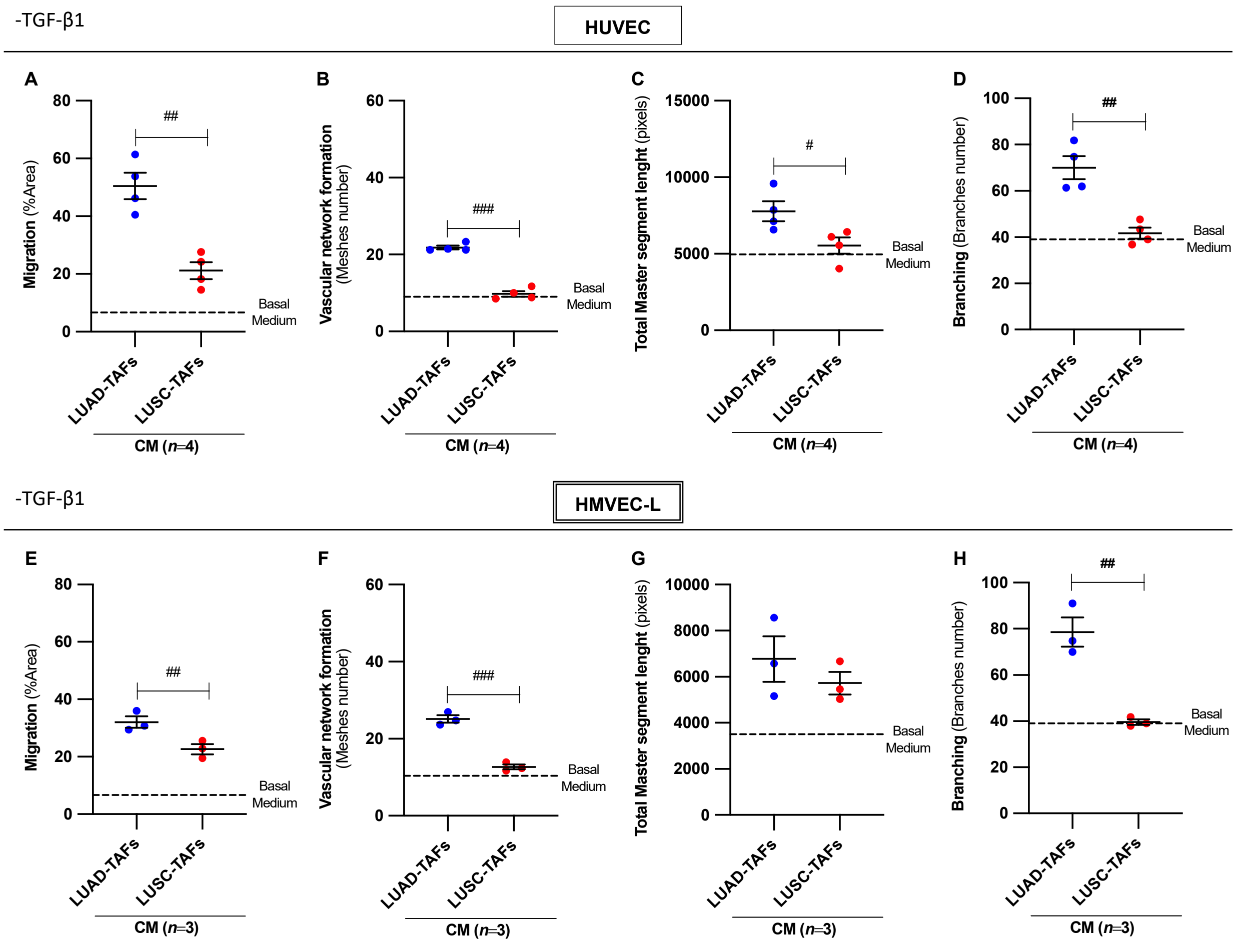

Supplementary Figure 4. **The basal conditioned medium (CM) of LUAD-TAFs promotes angiogenesis *in vitro* beyond LUSC-TAFs.** Pro-angiogenesis measurements were performed as in Fig 2C. (A-D) Average migration (A) and network descriptors (B-D) of HUVEC upon stimulation with CM from TAFs not pre-activated with exogenous TGF-β1. (E-H) Average migration (E) and network descriptors (F-H) of HMVEC-L stimulated in the same conditions. Horizontal dashed lines indicate the average values obtained upon stimulation with basal endothelial medium containing a minimal VEGF concentration (2 ng/ml). Error bars represent mean ± SEM. #, $p < 0.05$; ##, $p < 0.01$; ###, $p < 0.005$ comparing LUAD with LUSC.

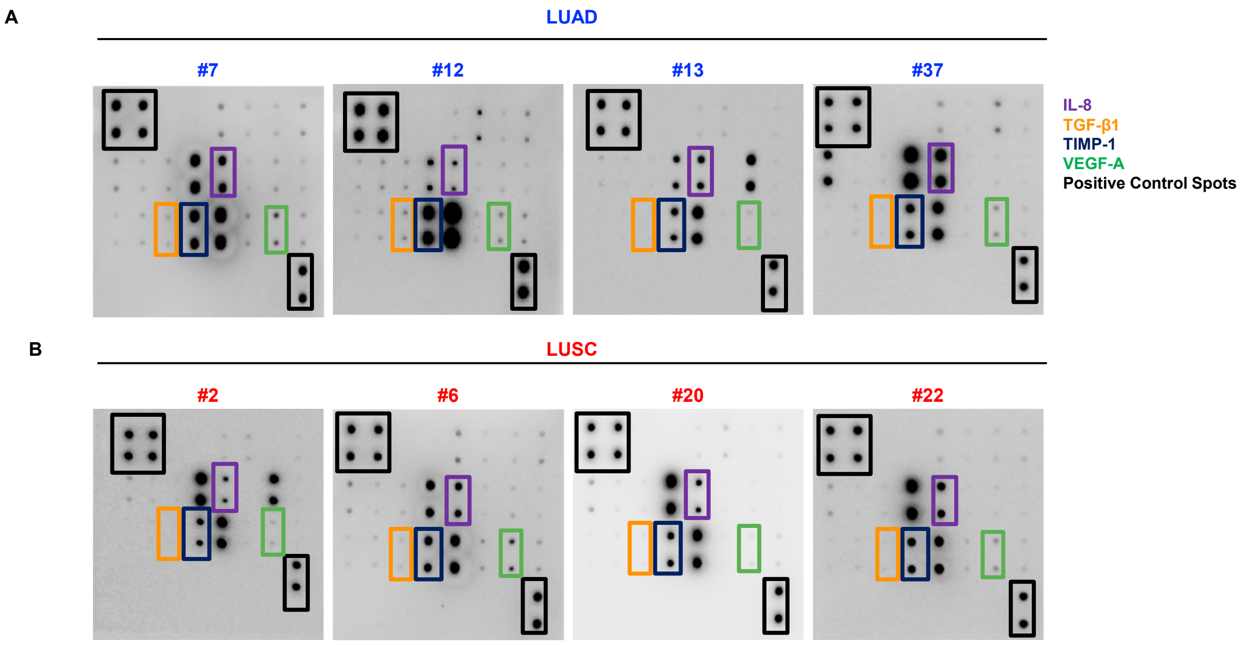

Supplementary Figure 5. **Differential secretion and expression of pro-angiogenic factors in LUAD-TAFs and LUSC-TAFs**. (A-B) Human angiogenesis antibody dot arrays analyzing 20 pro-angiogenic factors within the conditioned medium (CM) of a panel of LUAD-TAFs (A) and LUSC-TAFs (B). The pro-angiogenic factors that were differential expressed (CXCL-8, TGF-β1, TIMP-1 and VEGF-A) are indicated by the colored squares. Number after # indicates patient reference as in the main text.

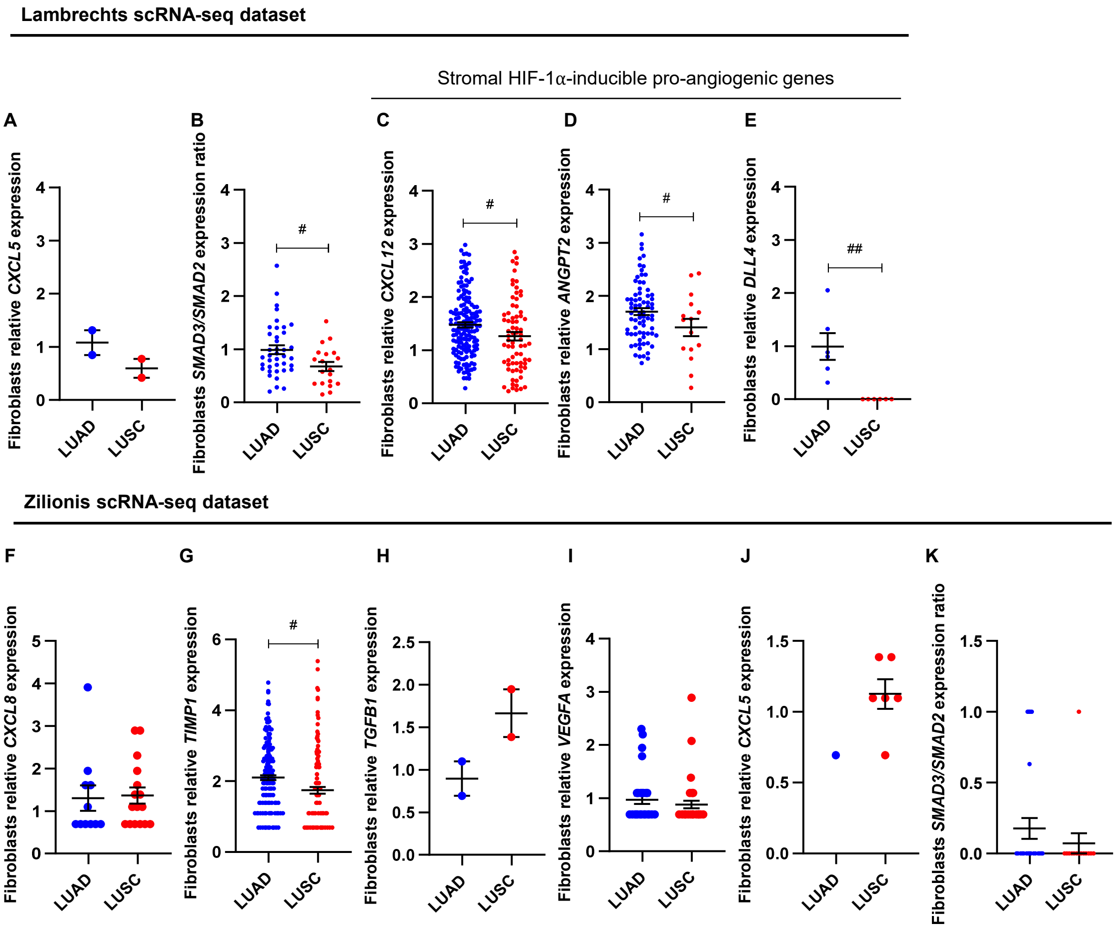

Supplementary Figure 6. LUAD-TAFs display distinct expression profiles of pro-angiogenic factors and HIF-dependent stromal factors compared to LUSC-TAFs. (A-E) Histotype-expression patterns in fibroblasts within the scRNA-seq Lambrechts dataset of *CXCL5* (A), the expression ratio of *SMAD3/SMAD2* (B), and the stromal HIF-inducible proangiogenic genes *CXCL12* (C), *ANGPT2* (D) and *DLL4* (E). (F-K) Histotype-expression patterns in fibroblasts within the scRNA-seq Zilionis dataset of *CXCL8* (F), *TIMP1* (G), *TGFB1* (H), *VEGFA* (I), *CXCL5* (J), and the expression ratio of *SMAD3/SMAD2* (K). Error bars represent mean ± SEM. #, $p < 0.05$; ##, $p < 0.01$ comparing LUAD with LUSC.

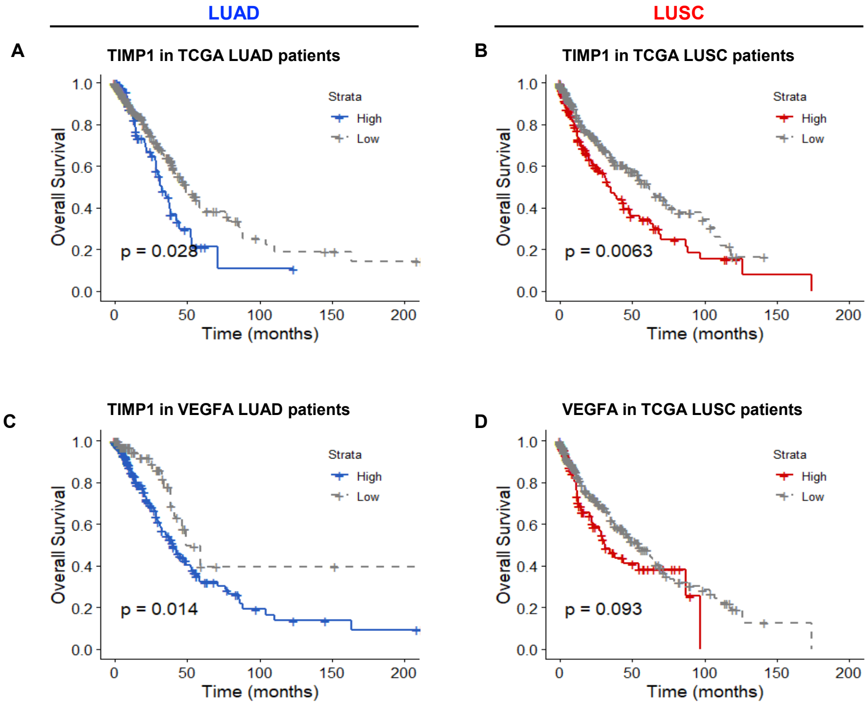

Supplementary Figure 7. Kaplan-Meier survival curves for *TIMP1* and *VEGFA* in LUAD and LUSC. (A-D) Kaplan-Meier survival curves stratifying patients according to *TIMP1* (A-B) and *VEGFA* (C-D) expression in LUAD (A,C) and LUSC (B,D) using data from the TCGA database. Survival curves were compared by log-rank test.

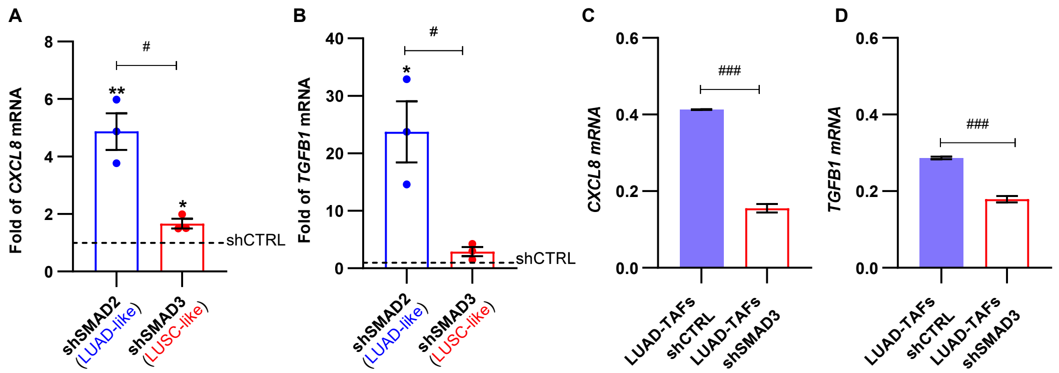

Supplementary Figure 8. Impact of SMAD2/3 in the expression of pro-angiogenic factors in fibroblasts. (A-B) Fold mRNA expression of the pro-angiogenic factors *CXCL8* (A) and*TGFB1* (B) in shSMAD2 or shSMAD3 CF^{hTERT} (#5) with respect to shCTRL CF^{hTERT} (#5). (C-D) mRNA expression of the pro-angiogenic factors *CXCL8* (C) and *TGFB1* (D) in a panel of LUAD-TAFs. (n=2: #12, #37) upon *SMAD3* knockdown by shRNA. Error bars represent mean ± SEM. #, $p < 0.05$; ###, $p < 0.005$; comparing shSMAD2 with shSMAD3 or LUAD-TAFs shControl with LUAD-TAFs shSMAD3. * $p < 0.05$; **, $p < 0.01$ comparing with shCTRL.

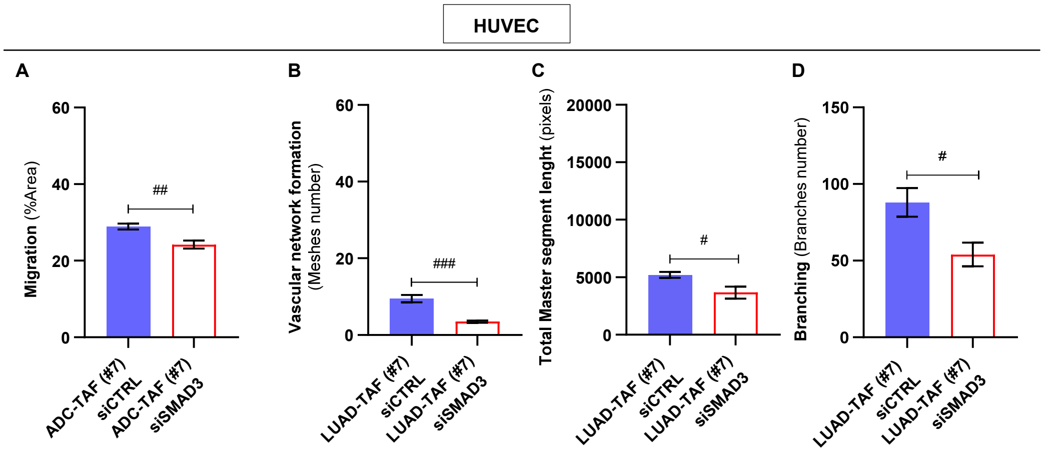

Supplementary Figure 9. LUADC-TAFs promotion of endothelial cell migration and angiogenesis is mediated by SMAD3 expression. (A) Endothelial cell migration in HUVEC upon stimulation with the CM of TGF-β1-activated siSMAD3 LUAD-TAF (#7) as described in Fig. 2C. (B-D) Endothelial cell network formation descriptors in HUVEC elicited by the concentrated CM of siSMAD3 LUAD-TAF (#7) as described in Fig. 2C, including number of meshes (B), total master segment length (C) and number of branches (D). Error bars represent mean ± SEM. #, $p < 0.05$; ##, $p < 0.01$; ###, $p < 0.005$ comparing siSMAD3 with or siControl (siCTRL).

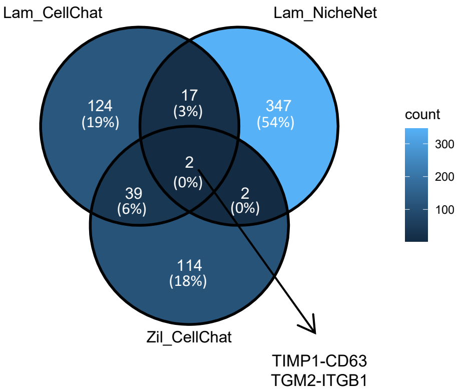

Supplementary Figure 10. Venn diagram comparing the outputs of the fibroblast-endothelial ligand-receptor pairs significantly upregulated in LUAD versus LUSC identified through the analysis of the two scRNA-seq datasets (Lambrechts, Zilionis) using two packages (CellChat, NicheNet). The comparison identified only 2 consensus ligand-receptor pairs: TIMP1-CD63 and TGM2-ITGB1.

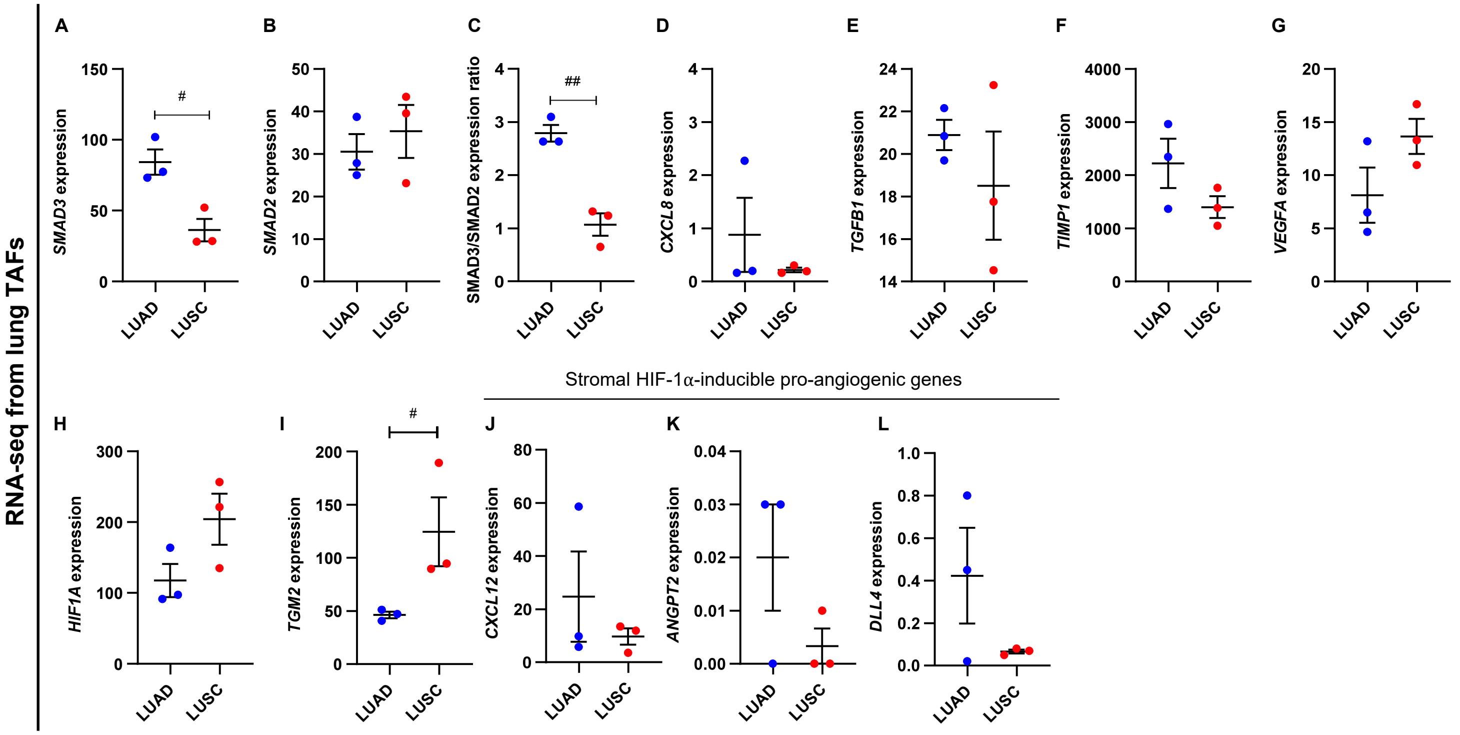

Supplementary Figure 11. Histotype-dependent expression patterns in lung TAFs. RNA-seq data showing the histotype-expression patterns of *SMAD3/SMAD2* ratio (A-C), *CXCL8* (D), *TGFB1* (E), *TIMP1* (F), *VEGFA* (G), *HIF1A* (H), *TGM2* (I), and stromal HIF-inducible proangiogenic genes *CXCL12* (J), *ANGPT2* (K) and *DLL4* (L). Error bars represent mean ± SEM. #, $p < 0.05$; ##, $p < 0.01$; comparing LUAD with LUSC.

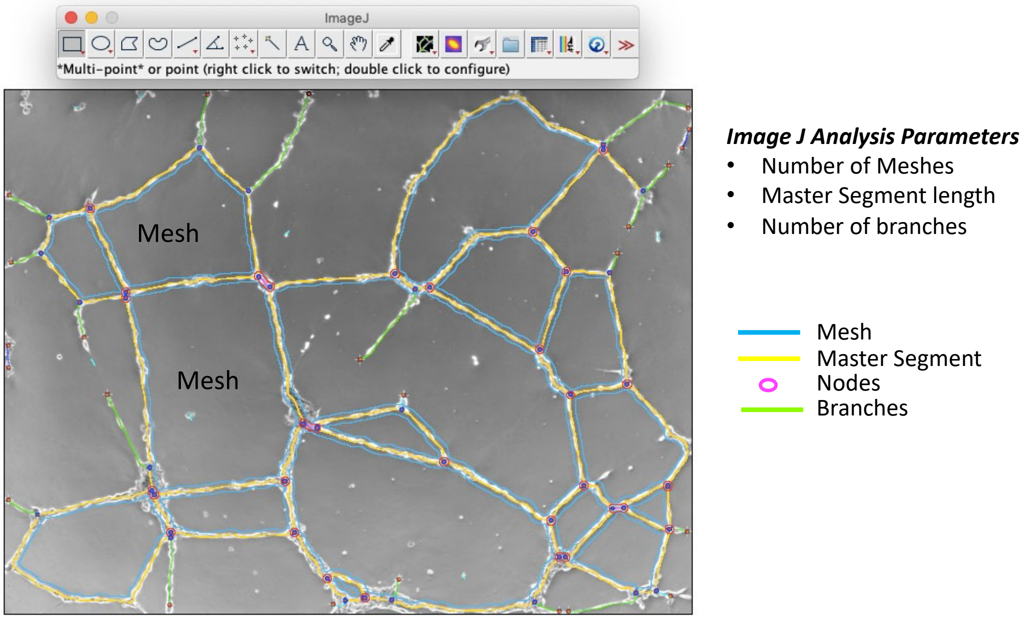

Supplementary Figure 12. Illustrative image of the network descriptors elicited by the Angiogenesis Analyzer plugin from Image J used to analyze the Endothelial Tube Formation Assay on Matrigel. $3x10^4$ HUVEC or HMVEC-L cells were seeded in 48 well coated with growth factor reduced Matrigel for 16h. Phase contrast images were analyzed using the Angiogenesis Analyzer plugin for image J (Carpentier et al, Sci Rep 2020), which provide the following pseudocolored network descriptors: number of meshes (cyan), master segment length (yellow), nodes (pink), branches (green).
