# Supplementary material for: Antagonistic SMAD2/3 control of TIMP-1, VEGF-A, and hypoxia signaling in myofibroblasts shapes histotype-specific angiogenesis in lung cancer
Source: Cell Death Dis. 2026 Mar 30;17(1):431. doi: 10.1038/s41419-026-08677-2 (PMC13156306; doi:10.1038/s41419-026-08677-2)
Supplement: Supplementary file 7 — Supplementary Table S4 [file 41419_2026_8677_MOESM7_ESM.pdf]

Antagonistic SMAD2/3 control of TIMP-1, VEGF-A, and hypoxia signaling in myofibroblasts shapes histotype-specific angiogenesis in lung cancer
***by* Díaz-Valdivia et al.**

Supplementary Table S4. Summary of clinicopathologic variables of the lung cancer patients from the CIBERES cohort used in the TMAs

Clinical variable	n (%)
Age (y.o.)	
< 65	90 (43.3%)
≥ 65	118 (56.7%)
Gender	
Female	30 (14.4%)
Male	178 (85.6%)
Race	
Caucasian	208 (100%)
Other	0
Smoking history	
Never	20 (9.6%)
Former	101 (48.6%)
Current	87 (41.8%)
Cardiovascular comorbidity	
No	118 (56.7%)
Yes	90 (43.3%)
Histologic subtype	
Adenocarcinoma	105 (50.2%)
Squamous cell carcinoma	92 (44.0%)
Large cell carcinoma	12 (5.7%)
Differentiation grade	
Well/mod. differentiated (G1,G2)	151 (74.8%)
Poorly differentiated (G3)	51 (25.2%)
Tumor stage (TNM)[a]	
IA1	6 (2.9%)
IA2	21 (10.2%)
IA3	31 (15.0%)
IB	47 (22.8%)
IIA	15 (7.3%)
IIB	56 (27.2%)
IIIA	29 (14.1%)
IVA	1 (0.5%)

NOTE: [a]According to 8th edition of the IASLC staging classification;
